# A heterozygous *LAMA5* variant may contribute to slowly progressive, vinculin-enhanced familial FSGS and pulmonary defects

**DOI:** 10.1172/jci.insight.158378

**Published:** 2022-12-08

**Authors:** Jun-Ya Kaimori, Yamato Kikkawa, Daisuke Motooka, Tomoko Namba-Hamano, Ayako Takuwa, Atsuko Okazaki, Kaori Kobayashi, Arisa Tanigawa, Yuko Kotani, Yoshihiro Uno, Kazuto Yoshimi, Koki Hattori, Yuta Asahina, Sachio Kajimoto, Yohei Doi, Tatsufumi Oka, Yusuke Sakaguchi, Tomoji Mashimo, Kiyotoshi Sekiguchi, Akihiro Nakaya, Motoyoshi Nomizu, Yoshitaka Isaka

**Affiliations:** 1 Department of Inter-Organ Communication Research in Kidney Diseases and; 2 Department of Nephrology, Osaka University Graduate School of Medicine, Osaka, Japan.; 3 Department of Clinical Biochemistry, Tokyo University of Pharmacy and Life Sciences, Tokyo, Japan.; 4 Genome Information Research Center, Research Institute for Microbial Diseases, and; 5 Immunology Frontier Research Center, Osaka University, Osaka, Japan.; 6 Department of Genome Informatics, Osaka University Graduate School of Medicine, Osaka, Japan.; 7 Diagnostics and Therapeutics of Intractable Diseases, Intractable Disease Research Center, Graduate School of Medicine, Juntendo University, Tokyo, Japan.; 8 Medical Solutions Division, NEC Corporation, Tokyo, Japan.; 9 Institute of Experimental Animal Sciences and; 10 Genome Editing Research and Development (R&D) Center, Osaka University Graduate School of Medicine, Osaka, Japan.; 11 Division of Animal Genetics, Laboratory Animal Research Center, The Institute of Medical Science;; 12 Division of Genome Engineering, Center for Experimental Medicine and Systems Biology, The Institute of Medical Science;; 13 Division of Matrixome Research and Application, Institute for Protein Research; and; 14 Laboratory of Genome Data Science, Graduate School of Frontier Sciences, The University of Tokyo, Tokyo, Japan.

**Keywords:** Genetics, Nephrology, Extracellular matrix, Genetic diseases

## Abstract

The *LAMA5* gene encodes laminin α5, an indispensable component of glomerular basement membrane and other types of basement membrane. A homozygous pathological variant in *LAMA5* is known to cause a systemic developmental syndrome including glomerulopathy. However, the roles of heterozygous *LAMA5* gene variants in human renal and systemic diseases have remained unclear. We performed whole-exome sequencing analyses of a family with slowly progressive nephropathy associated with hereditary focal segmental glomerulosclerosis, and we identified what we believe to be a novel probable pathogenic variant of *LAMA5*, NP_005551.3:p.Val3687Met. In vitro analyses revealed cell type–dependent changes in secretion of variant laminin α5 laminin globular 4-5 (LG4-5) domain. Heterozygous and homozygous knockin mice with a corresponding variant of human *LAMA5*, p.Val3687Met, developed focal segmental glomerulosclerosis–like pathology with reduced laminin α5 and increased glomerular vinculin levels, which suggested that impaired cell adhesion may underlie this glomerulopathy. We also identified pulmonary defects such as bronchial deformity and alveolar dilation. Reexaminations of the family revealed phenotypes compatible with reduced laminin α5 and increased vinculin levels in affected tissues. Thus, the heterozygous p.Val3687Met variant may cause a new syndromic nephropathy with focal segmental glomerulosclerosis through possibly defective secretion of laminin α5. Enhanced vinculin may be a useful disease marker.

## Introduction

In the kidney, the glomerular basement membrane (GBM) is a sheet-like extracellular matrix meshwork that separates 2 cellular layers: endothelial cells and podocytes. The GBM plays critical roles in filtration and the maintenance of glomerular morphology (1). The basement membrane (BM) is typically composed of matrix proteins: laminin, collagen IV, nidogen, and sulfated proteoglycan (2). Laminins are typically present as heterotrimeric glycoproteins composed of α, β, and γ chains. The GBM contains an atypical assortment that includes a laminin isoform, laminin-521 (LM-521), also known as α5β2γ1 (2).

Laminin’s α chains serve as chief components of scaffolds for cell-matrix adhesion by functioning as direct ligands for integrins (3). Cell-matrix adhesions act as sites of force transmission and connectors between BM and the actin cytoskeleton. Cell-matrix adhesions comprise multiple proteins, including integrin, focal adhesion kinase, and vinculin (4).

Among several laminin α chains, laminin α5 is a major α chain that is widely expressed in fetal and adult tissues (5). During glomerulogenesis, laminin composition changes from LM-111 (α1β1γ1) in early glomerular stages to LM-521 in the mature GBM (6). *Lama5*^−/−^ mice die at late fetal stages with multiple developmental defects (7), kidneys are small and occasionally absent in *Lama5*^−/−^ embryos, and the absence of laminin α5 in GBM prevents vascularization during glomerulogenesis (8). In addition, podocyte-specific inactivation of *Lama5* in mice causes varying degrees of proteinuria, indicating that podocyte-derived laminin α5 is required for the integrity of glomerular filtration (9). Lung epithelial cell–specific *Lama5*^−/−^ mice exhibit dilated, enlarged airspaces (10). Recently, Falcone et al. identified a homozygous mutation (E884G) in *Lama5* by mouse phenotype-driven mutagenesis screening. The mice homozygous for the E884G *Lama5* variant developed severe proteinuria (11). These lines of evidence in experiments using *Lama5* gene-modified mice indicate that laminin α5 plays pivotal roles in GBM and lung BM.

Despite a large body of evidence from basic studies showing the importance of laminin α5 in kidney and lung, clinical clues about it have been very scarce. The clinical importance of laminin β2 has already been shown by the discovery of Pierson syndrome, a congenital nephrotic syndrome (NS) with eye and neuronal defects (12, 13), which are caused by variants in the laminin β2 subunit that influence the synthesis or function of LM-521. Recently, after the initial identification of homozygous *LAMA5* pathogenic variants in pediatric patients with NS (14), a detailed report of a homozygous *LAMA5* variant demonstrated that the affected patient had a developmental disease involving multiple organ systems, which included focal segmental glomerulosclerosis (FSGS) (15). Furthermore, various reports of compound heterozygous *LAMA5* variants showed that patients had NS and some reached end-stage renal disease (16, 17). However, the importance of heterozygous *LAMA5* variants in human NS and kidney disease has remained unclear; a heterozygous *LAMA5* variant has not been definitively found to cause monogenic human kidney disease, including NS or FSGS. In previous reports, *LAMA5* G3685R was identified as a “probable pathogenic gene variant for heterozygous FSGS” (18, 19). However, *LAMA5* G3685R is evaluated as “likely benign” or “benign” in the Clinvar website (https://www.ncbi.nlm.nih.gov/clinvar/) and the allele frequency of G3685R (60884427C>T) is 0.01239 in the Genome Aggregation Database (gnomAD) (European, non-Finnish), suggesting that G3685R is not a rare variant in Europe. These facts raise doubts about whether *LAMA5* G3685R is a heterozygous *LAMA5* variant responsible for familial nephropathy with FSGS.

Here, we report that a newly discovered heterozygous variant in *LAMA5* (GenBank O15230) might cause slowly progressive FSGS and lung structural defects, possibly inducing chest pain. The variant was associated with a reduction of laminin α5 levels in BM within these tissues, leading to increased expression of the mechanosensory protein vinculin in cell-matrix adhesions.

## Results

### Case study.

**Figure 1:**
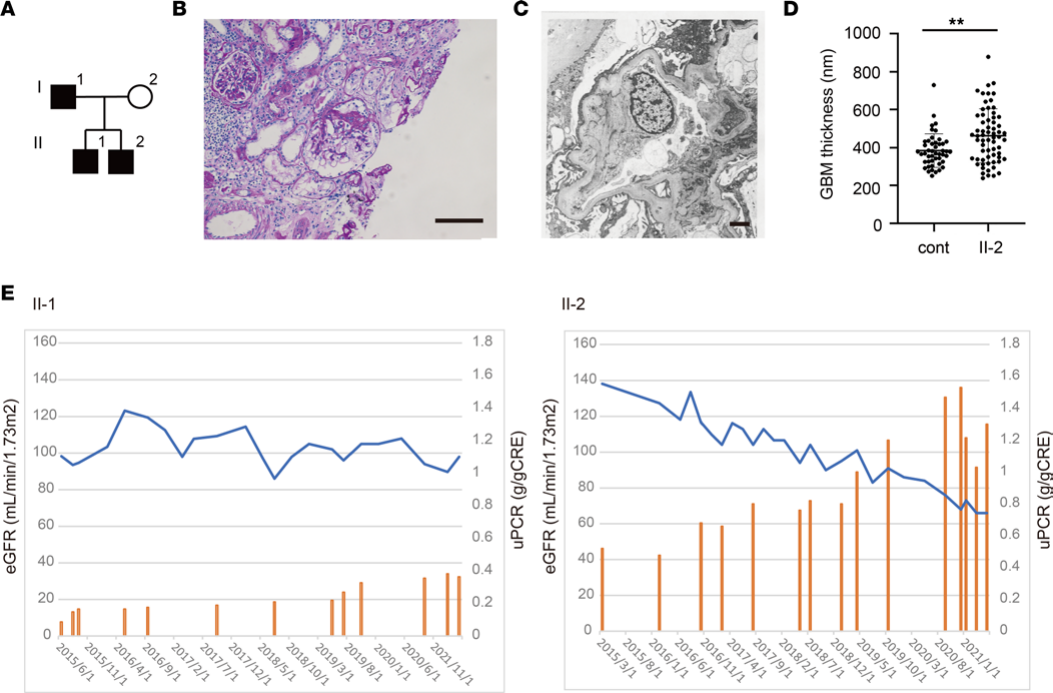
Clinical analysis of a family with hereditary FSGS. (**A**) Pedigree of family with hereditary FSGS. (**B**) Periodic acid–Schiff staining images of renal biopsy sample from patient I-1. Scale bar: 100 μm. (**C**) Electron microscopy image of GBM in patient II-2. Scale bar: 2 μm. (**D**) GBM thickness in patient II-2. **P < 0.01; Mann-Whitney *U* test. Cont, data from a similarly aged, sex-matched patient with minimal-change NS. (**E**) Clinical courses of patients II-1 and II-2 in the past 5 years. Blue lines represent estimated glomerular filtration rate (eGFR). Yellow bars show urine protein/creatinine ratio (uPCR).

The pedigree of the Asian family in this study is shown in Figure 1A. Patients with renal problems are indicated in black. Patient I-1 (the father) had exhibited proteinuria at the age of 16 years; he had been diagnosed with glomerulopathy with FSGS at the age of 23 years on the basis of renal biopsy findings (Figure 1B). His disease progressed to end-stage renal disease; he began continuous peritoneal dialysis at the age of 44 years after approximately 20 years of stable proteinuria and renal function. Patient II-2 exhibited urine dipstick 2+ proteinuria at 14 years of age; he was diagnosed with early nephropathy associated with FSGS with positive effacement of podocyte foot processes (Figure 1C) and thicker GBM than a similarly aged, sex-matched patient with minimal-change NS (control) (Figure 1D). His renal function has been gradually decreasing with increasing proteinuria for the last 5 years (Figure 1E). Patient II-1 exhibited urine dipstick 2+ proteinuria at the age of 19 years and has shown no abnormalities in renal biopsy tissues. He has maintained renal function with urine dipstick 2+ proteinuria thus far (Figure 1E). Patients II-1 and II-2 did not demonstrate any urine occult blood. All 3 patients experienced chest pain with normal ECG and echocardiography findings. Patient I-1 also had persistent sputum and coughing for 30 years. Notably, the chest pain could be relieved by deep breathing in, suggesting that the chest pain did not originate from pleura. The affected family members demonstrated no kidney dysplasia or vesicoureteral reflux, which were previously identified in a patient homozygous for *LAMA5* (15). In other family members, no renal abnormalities were observed.

### Next-generation sequencing–driven quad exome analyses.

**Figure 2:**
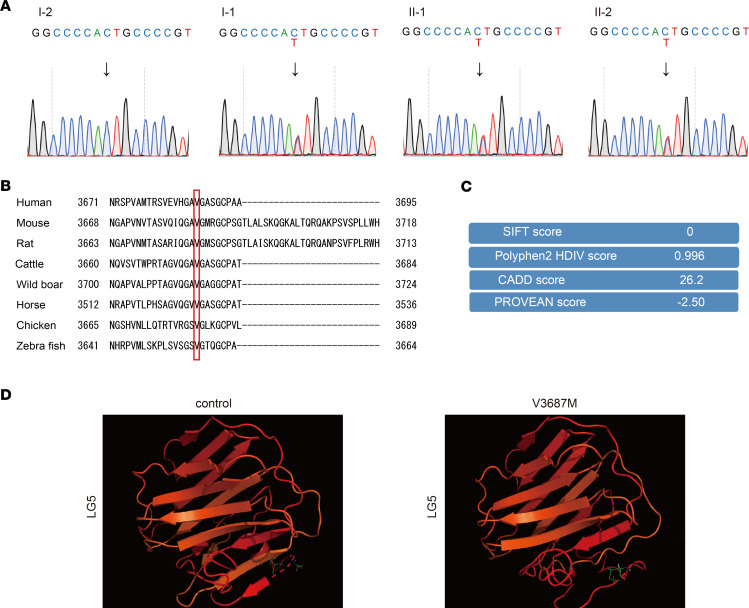
Identification of a potentially novel *LAMA5* heterozygous variant for familial FSGS. (**A**) Sanger sequence chromatograms of the patients’ family members. Arrows in chromatograms indicate mutated sites. (**B**) Amino acid sequence alignment of laminin α5 proteins from multiple species. (**C**) Scores of variant pathogenicity by SIFT, Polyphen2, CADD, and PROVEAN. (**D**) Predicted 3D structures of control and variant V3687M laminin α5 LG5 modules. The amino acid molecular models indicated the positions Val3687 (control) and Val3687 (V3687M), respectively.

To identify the gene causing nephropathy with FSGS in this family, we conducted analyses of the genomic DNA sequences (Supplemental Table 1; supplemental material available online with this article; https://doi.org/10.1172/jci.insight.158378DS1) of family members shown in the pedigree (Figure 1A). Our analysis pipeline identified 54 possible pathogenic variants in 49 genes (Supplemental Table 2). Among these candidate genes, we narrowed this set down to 10 possible candidate genes with a combined annotation dependent depletion (CADD) score greater than 10 (*MAST2*, *MTMR9*, *RBM20*, *PTPRE*, *DGKH*, *SPTBN5*, *SEMA6B*, *NKX2-2*, *RIPOR3*, and *LAMA5*). Only *LAMA5* was associated with glomerulopathy with FSGS among these 10 genes. In the *LAMA5* gene, we identified the following variant: NC_000020.11:g.60884421C>T, NP_005551.3:p.Val3687Met (hereinafter referred to as *LAMA5* V3687M). This variant has not yet been reported in gnomAD and is only reported in 4.7KJPN (0.0006) and the Human Genetic Variation Database (HGVD) (0.000885). Using Sanger sequencing, we confirmed that the heterozygous variant was present in the patients’ genomes, but not in a healthy control (Figure 2A). Amino acid alignment analyses of laminin α5 in many species revealed that Val3687 was highly conserved and located in the C-terminal region of the protein, the laminin globular 5 (LG5) module (Figure 2B). This variant was assumed to negatively influence the laminin α5 molecular structure (Figure 2C). However, based on 3-dimensional (3D) structural prediction of the variant laminin α5 LG1–5 protein (20–22), these variants did not cause extensive changes in the predicted 3D arrangement of the LG1–5 module (Supplemental Figure 1); they only affected portions of the predicted β-sheet structure in the LG5 domain (Figure 2D).

### In vitro analyses of laminin α5 protein expression and secretion.

**Figure 3:**
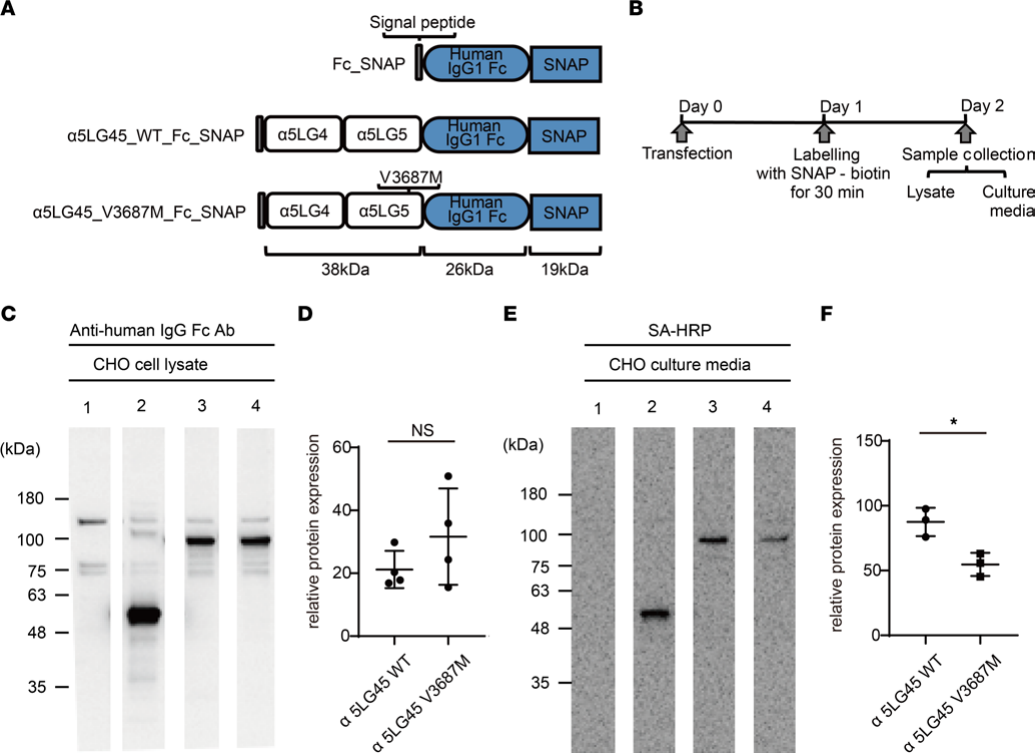
The fate of this variant laminin α5 protein in vitro. (**A**) Structures of transfected expression vectors. (**B**) Study design for this in vitro experiment. (**C**) IB analysis of variant laminin α5LG4–5 fragment/Fc-SNAP fusion proteins in lysates of CHO-K1 cells. Cell lysates of negative control (lane 1), Fc-SNAP control (lane 2), WT (lane 3), and V3687M (lane 4) were used for IB analysis. Recombinant proteins were detected using anti-human IgG Fc Ab and quantified by densitometry, as described in the Methods. (**D**) Density of Fc-SNAP band was defined as 100%. Each column represents the mean of triplicate assays. Bars show SDs. Both variant and WT proteins were expressed. Student’s *t* test. (**E**) Pulse-chase analysis of variant laminin α5LG4–5 fragment/Fc-SNAP fusion proteins secreted from CHO-K1 cells. Transfected cells were labeled with SNAP-Biotin for 30 minutes, then cultured in serum-free medium for 1 day. Biotinylated proteins in the conditioned media were visualized and quantified. (**F**) Density of biotinylated Fc-SNAP band was defined as 100%. *P < 0.05; Student’s *t* test. In CHO-K1 cells, the secretion of both variant proteins was significantly lower than the secretion of WT protein.

To clarify the extent to which laminin α5 protein fate is affected by the gene variant identified in our patients, we generated constructs of WT or the variant V3687M laminin α5 LG4–5 module conjugated to a human IgG1 Fc peptide and SNAP tag (Figure 3A). These constructs were transfected into CHO-K1 cells, and cell lysates were then analyzed by IB. Expression levels did not differ between WT protein and the variant LG4–5 module in this cell line (Figure 3, C and D). To determine the fate of the secreted protein, we conducted a pulse-chase assay with variant LG4–5 proteins by using the SNAP tag system. WT and the variant LG4–5 proteins with the SNAP tag were labeled with cell-permeable SNAP-biotin 1 day after transfection of expression vectors into CHO-K1 cells. At 2 days after transfection, we quantified labeled LG4–5 proteins in the cell culture media (Figure 3B). We discovered that the secretion of labeled variant proteins was reduced in the CHO-K1 cell media, compared with the level of WT protein (Figure 3, E and F); however, full-length variant laminin α5 proteins (mouse *Lama5*_V3684M) were hypothesized to form large heterotrimeric protein complexes with laminin β2 and laminin γ1 (Supplemental Figure 2). In the full-length variant laminin α5 protein experiments, we had only HEK293 cells stably expressing laminin β2 and laminin γ1, not CHO-K1 cells. We could not find a difference in the secreted quantity of heterotrimeric protein complexes including WT and variant proteins in HEK293 cells (Supplemental Figure 2). Our data revealed that the secretion of variant LG4–5 proteins might have been hindered when these proteins were expressed in CHO-K1 cells.

### Elucidation of renal phenotypes in heterozygous and homozygous Lama5-V3684M KI mice.

**Figure 4:**
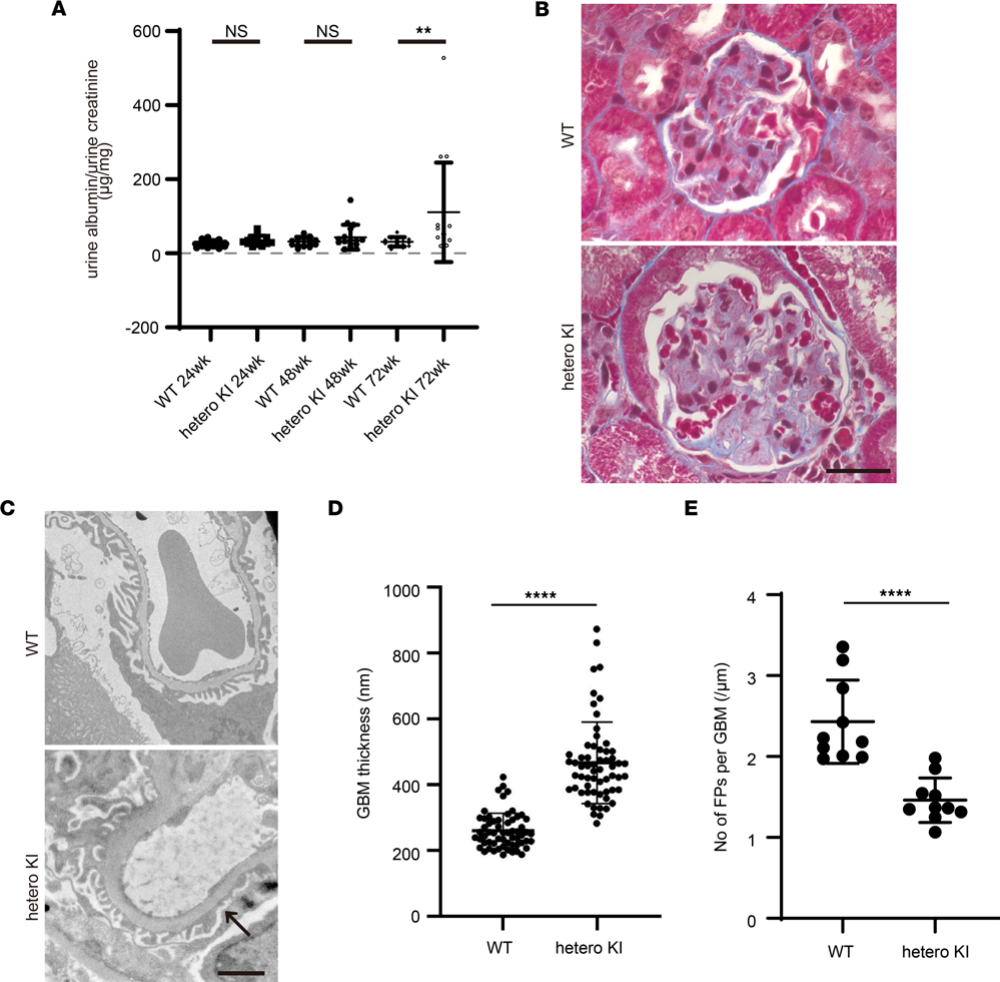
Renal phenotypes of heterozygous V3684M KI mice at 72 weeks of age. (**A**) Urine albumin excretion (urine albumin/creatinine ratio) of WT and heterozygous V3684M KI mice at 24, 48, and 72 weeks of age. **P < 0.01; Mann-Whitney *U* test. (**B**) Masson’s trichrome staining images of kidney tissues from WT and V3684M KI mice. Scale bar: 20 μm. (**C**) Electron micrographs of GBM from WT and heterozygous KI mice. Scale bar: 2 μm. The arrow indicates partial effacement of podocyte foot processes. (**D**) GBM thickness of WT and heterozygous V3684M KI mice at 72 weeks of age. ****P < 0.0001; Mann-Whitney *U* test. (**E**) Number of foot processes of podocyte per micrometer GBM length from WT and heterozygous V3684M KI mice at 72 weeks of age. ****P < 0.0001; Mann-Whitney *U* test.

**Figure 5:**
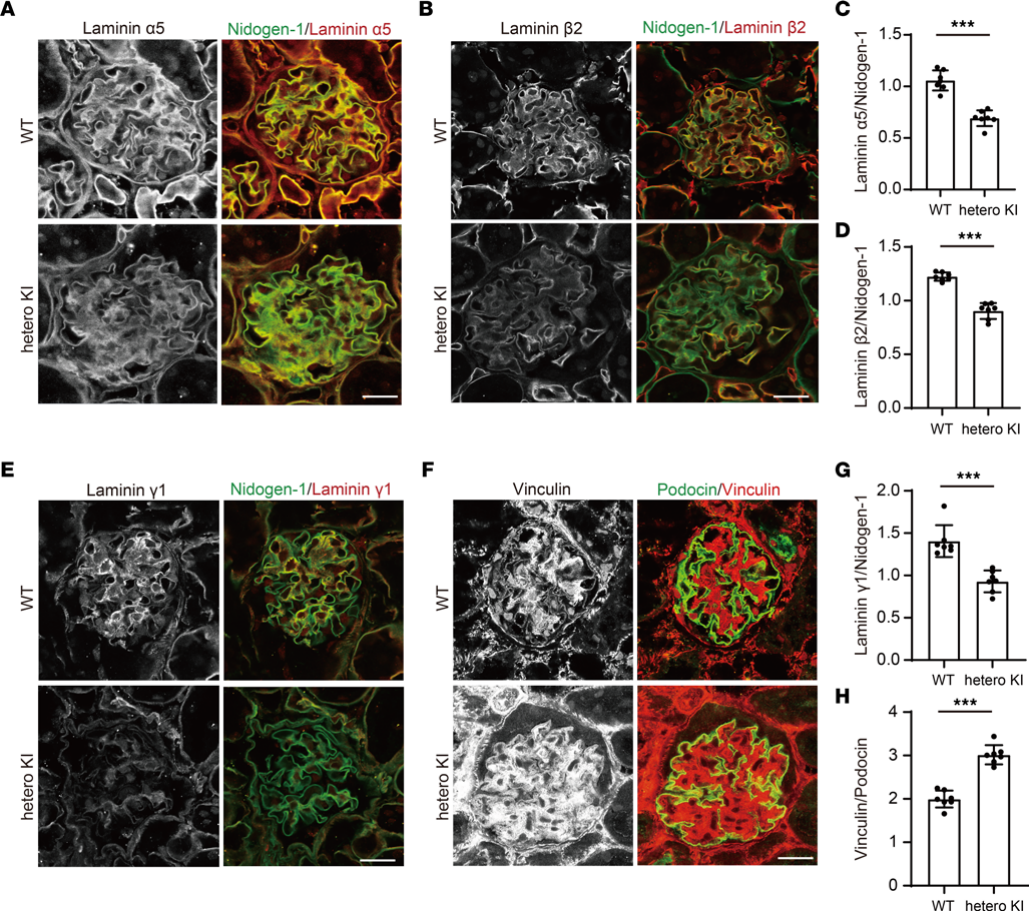
Protein expression of laminin α5, β2, γ1, and vinculin in the glomeruli in heterozygous V3684M KI mice at 72 weeks of age. Immunofluorescence staining images of laminin α5 (**A**), β2 (**B**), γ1 (**E**), and nidogen-1 in kidney glomeruli from WT and heterozygous KI mice. Scale bar: 20 μm. Quantification of laminin α5 (**C**), β2 (**D**), and γ1 (**G**) relative to nidogen-1 intensity in the glomerular area. Mean fluorescence intensity of each stain was calculated by Zen software. ***P < 0.001; Mann-Whitney *U* test. (**F**) Immunofluorescence staining images of podocin and vinculin in kidney glomeruli from WT and heterozygous KI mice. Scale bar: 20 μm. The tissues were fixed in acetone before the incubation with primary Abs. (**H**) Quantification of vinculin relative to podocin intensity in the glomerular area. ***P < 0.001; Mann-Whitney *U* test.

**Figure 6:**
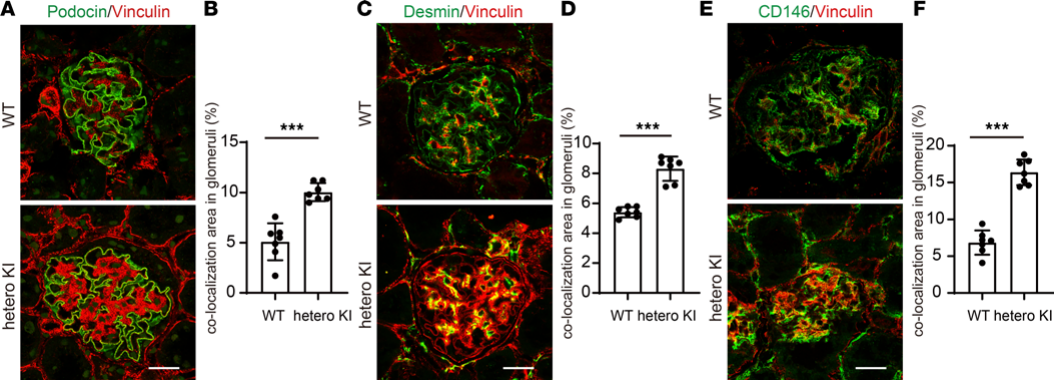
Increased vinculin localization in various glomerular components of heterozygous V3684M KI mice at 72 weeks of age. (**A**) Immunofluorescence staining images of podocin and vinculin in kidney glomeruli from WT and heterozygous KI mice. Scale bar: 20 μm. No fixation was performed before the incubation with primary Abs. (**B**) Quantification of vinculin and podocin colocalization area per glomerular area. ***P < 0.001; Mann-Whitney *U* test. (**C**) Immunofluorescence staining images of desmin and vinculin in kidney glomeruli from WT and heterozygous KI mice. Scale bar: 20 μm. (**D**) Quantification of vinculin and desmin colocalization area per glomerular area. ***P < 0.001; Mann-Whitney *U* test. (**E**) Immunofluorescence staining images of CD146 and vinculin in kidney glomeruli from WT and heterozygous KI mice. Scale bar: 20 μm. (**F**) Quantification of CD146 and desmin colocalization area per glomerular area. ***P < 0.001; Mann-Whitney *U* test.

Isolation of the *LAMA5* variant, NC_000020.11:g.60884421C>T, as a gene variant responsible for hereditary glomerulopathy with FSGS was only based on this single family. To confirm whether this variant could cause the phenotypes observed in this family, we generated knockin (KI) mice expressing a mouse-compatible version of the variant laminin α5 protein produced by this family (*Lama5*-V3684M). To more closely investigate the pathophysiology in these patients, we examined the phenotypes of heterozygous animals. Until 48 weeks of age, they displayed proteinuria similar to the level observed in WT animals; however, at 72 weeks of age, they showed substantial proteinuria (Figure 4A). The mice showed FSGS-like pathological changes (including partial podocyte foot process effacement; Figure 4, B and C) at 72 weeks of age. The heterozygous KI mice also demonstrated homogeneous and thicker GBM and fewer foot processes of podocytes per GBM length than WT (Figure 4, C–E). Consistent with the in vitro findings demonstrating compromised variant LG4–5 protein secretion, the expression of laminin α5 protein in GBM was greatly reduced (Figure 5, A and C), whereas the level of a GBM marker, nidogen-1, did not change. We used an anti-laminin α5 Ab (Supplemental Table 4) that recognizes parts of the laminin α5 protein distinct from LG domains (LEb/L4b). We also identified that the levels of laminin β2 and laminin γ1, the other components of LM521, were significantly reduced (Figure 5, B, D, E, and G). Data indicating reduced levels of scaffolding protein complex, LM521, prompted us to focus on mechanosensory markers in cell-matrix adhesions, vinculin, and phosphorylated focal adhesion kinase (p-FAK). The expression of a podocyte maturation marker (podocin) did not change, whereas the expression of vinculin was greatly enhanced (Figure 5, F and H). Interestingly, the vinculin staining was colocalized with markers of podocytes (podocin), mesangial cells (desmin), and endothelial cells (CD146). The degree of vinculin colocalization with all of these markers was increased in heterozygous KI mouse glomeruli (Figure 6, A–F). p-FAK immunostaining in glomeruli did not differ between heterozygous KI mice and WT mice (Supplemental Figure 3). These findings indicated that mice with heterozygous variant V3684M could develop nephropathy with FSGS, which affected the adhesion of many cell types in the glomeruli. The changes in the glomerular expression of laminin α5 and vinculin were also observed in the peritubular area of heterozygous KI mice. In the immunofluorescence staining of other chains of laminin, other components of the extracellular matrix (ECM), and receptors for laminin α5, we identified the increased expression of collagen I and fibulin 2 proteins and the decreased expression of integrin β1 in the glomeruli of heterozygous KI mice (Supplemental Figure 4, A–C). In addition, in the homozygous KI mice at 16 weeks of age, we identified FSGS-like phenotypes and virtually the same findings as in heterozygous KI mice (Supplemental Figure 5, A–E; Supplemental Figure 6, A–H; and Supplemental Figure 7, A–F), suggesting that the dose of this variant allele affects the severity of KI mouse phenotypes. These KI mouse data suggested that the potentially novel heterozygous *LAMA5* variant V3687M is so potent that it could cause FSGS-like disease across species boundaries.

### Elucidation of systemic phenotypes in heterozygous and homozygous Lama5-V3684M KI mice.

**Figure 7:**
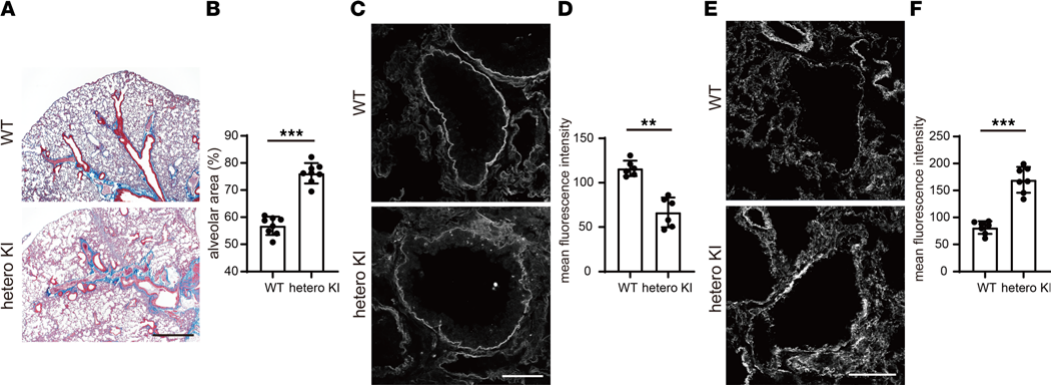
Extrarenal phenotypes of heterozygous V3684M KI mice at 72 weeks of age. (**A**) Lower magnified images of Masson’s trichrome staining of lung tissues from WT and heterozygous V3684M KI mice. Bronchial tube deformities and enlarged alveolar area were observed in the lung tissue from heterozygous KI mice at 72 weeks of age. Scale bar: 1,000 μm. (**B**) Quantification of alveolar area in lung tissues from WT and heterozygous KI mice. Alveolar area was quantified using ImageJ software (NIH). ***P < 0.001; Mann-Whitney *U* test. (**C**) Immunofluorescence staining images of laminin α5 in BMs in lung bronchial tubes from WT and heterozygous KI mice at 72 weeks of age. Laminin α5 staining was greatly reduced in BM from heterozygous KI mice. Scale bar: 50 μm. (**D**) Quantification of mean laminin α5 fluorescence intensity along BM from WT and heterozygous KI mice at 72 weeks of age. Mean laminin α5 fluorescence intensity along BM was calculated using Zen software. **P < 0.01; Mann-Whitney *U* test. (**E**) Immunofluorescence staining images of vinculin in BM of lung bronchial tubes from WT and heterozygous KI mice at 72 weeks of age. Vinculin staining was greatly enhanced in BM from heterozygous KI mice. Scale bar: 50 μm. (**F**) Quantification of mean vinculin fluorescence intensity along BM from WT and heterozygous KI mice at 72 weeks of age. ***P < 0.001; Mann-Whitney *U* test.

In heterozygous KI mice, distinct phenotypes were observed in the lung, including thick and deformed bronchial tubes, as well as dilated alveolar areas similar to emphysema (Figure 7, A and B). Notably, remarkable loss of laminin α5 expression was observed in the BM of bronchial tubes and around tissue areas (Figure 7, C and D). Furthermore, considerably enhanced expression of vinculin protein was observed along the BM and around tissue areas (Figure 7, E and F; Supplemental Figure 8). These findings suggested characteristic structural defects in these tissues, possibly caused by the reduction of laminin α5 protein in BM. In the analyses of homozygous KI mice, we identified other phenotypes (dilated tubules in the kidney and dilated veins in the liver) in addition to the structural defects in the lung at 16 weeks of age (Supplemental Figure 9, A–C).

### Analysis of variant laminin α5 and vinculin detection in patient glomeruli.

**Figure 8:**
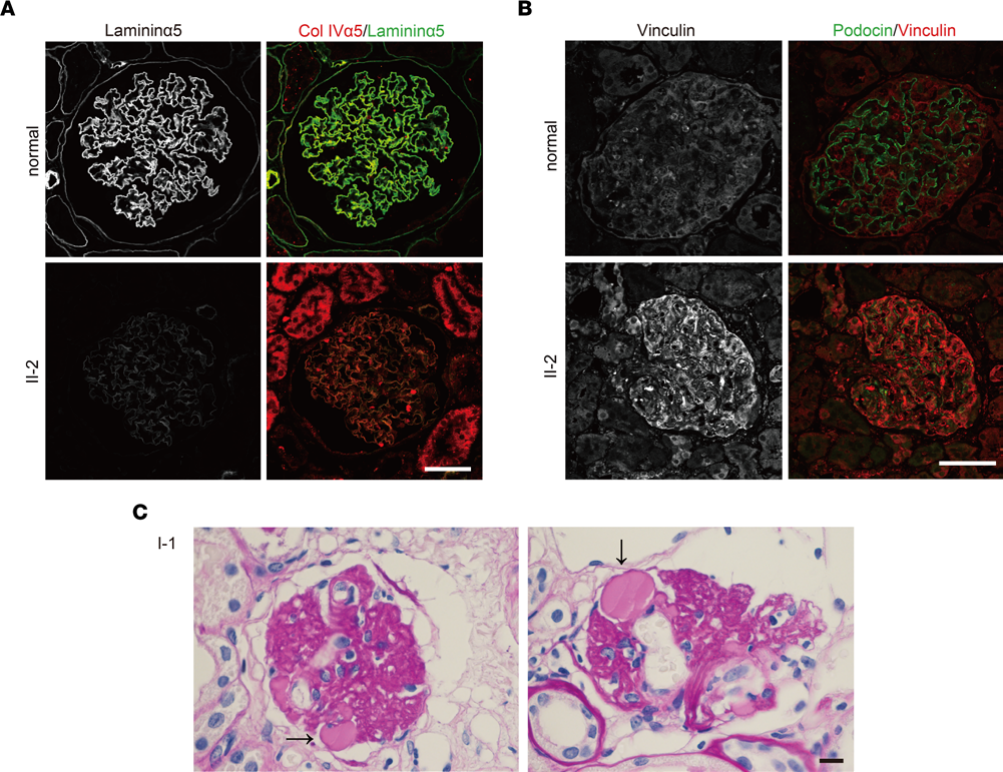
Renal phenotypes of patients. (**A**) Immunofluorescence staining images of laminin α5 and α5 chain of type IV collagen in glomeruli from a healthy control volunteer and from patients II-1 and II-2. Laminin α5 staining in GBM was lower in patients than in the healthy control. Scale bar: 50 μm. (**B**) Immunofluorescence staining images of a mechanosensory protein, vinculin, and a podocyte marker, podocin, in glomeruli from a healthy control volunteer and from patient II-2. Vinculin staining was greatly enhanced in podocin-positive cells and endothelial cells in patient II-2. Scale bar: 50 μm. (**C**) Periodic acid–Schiff staining image of kidney tissue from patient I-1. Glomerular endothelial cell detachments were observed. Scale bar: 50 μm. Arrows indicate the area of endothelial cell detachments.

Following acquisition of the data obtained via the KI mice, we performed reexaminations of the 3 members described above. We conducted IHC analyses using renal tissue sections from patients to confirm that the variant protein led to reduced laminin α5 and enhanced staining of vinculin in glomeruli. Before these analyses, we confirmed that the mouse monoclonal anti-laminin α5 Ab (mAb 5D6) recognized the LM511-E8 protein domain, rather than the α5LG4–5 modules where the variant is located (Supplemental Figure 10, A and B). Immunofluorescence staining using mAb 5D6 revealed that laminin α5 protein expression was greatly reduced in patient II-2, consistent with the results in heterozygous KI V3684M mice (Figure 8A). Importantly, the expression of vinculin was greatly enhanced in patient II-2’s tissue (Figure 8B), with reduced expression of the podocyte maturation marker podocin. Compatible with the notion that cell adhesion to GBM might be affected in this dis-ease, endothelial cell detachment was observed in kidney tissue from patient I-1 (Figure 8C).

### Detailed characterization of patient lung phenotypes.

**Figure 9:**
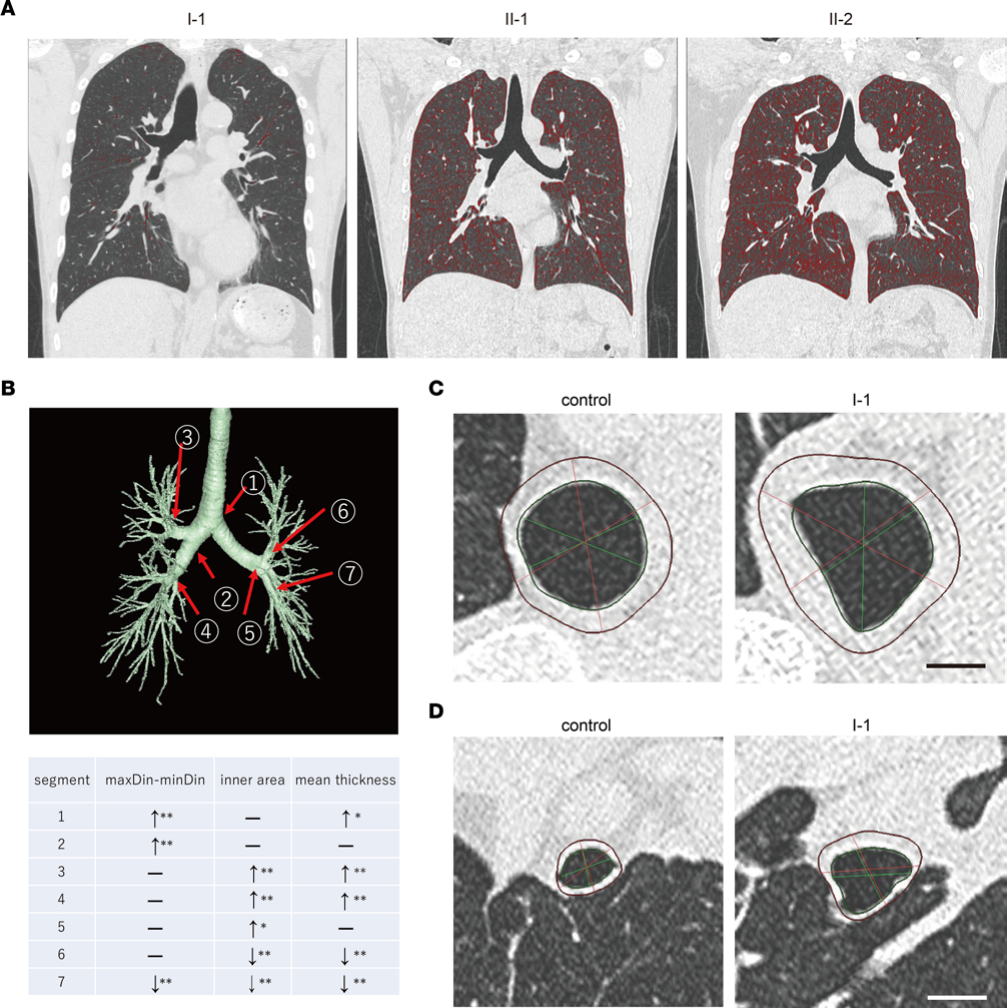
Extrarenal phenotypes of patients. (**A**) Emphysematous changes in patients. Low-attenuation areas (LAAs) were calculated under the threshold of –950 Hounsfield Units and are indicated in red in each chest CT image. Patients II-1 and II-2 had slight and moderate emphysematous changes in their lungs, respectively. Patient I-1 had no emphysematous change. (**B**) Detailed 3D analyses of bronchial tubes using chest CT. 3D bronchial tree of patient I-1 with indicated measured segments and analysis results. maxDin-minDin: difference between maximum inner diameter and minimum inner diameter at each segment. *P < 0.05; **P < 0.01; Mann-Whitney *U* test. –, not significant. *↑*, increase; ↓, decrease. (**C**) Cross-sectional images of main bronchi (segment 1) of patient I-1 and an age- and sex-matched healthy control volunteer showing a deformed and thickened bronchus in patient I-1. Scale bar: 1 cm. (**D**) Cross-sectional images of bronchial tubes (segment 3) of the healthy control volunteer and patient I-1 showing lost bronchial tubes, as well as thickened bronchial tubes, in patient I-1. Scale bar: 1 cm.

On the basis of the findings in heterozygous V3684M KI mice, we conducted detailed examinations of our patients to identify potential extrarenal phenotypes. Patient I-1 had exhibited persistent sputum and coughing for almost 30 years, while all 3 patients exhibited nonpleuritic chest pain. Thus, we suspected that these patients might have lung structural defects because 25% of patients with bronchiectasis have chest pain, which is nonpleuritic in most of these patients (23). Detailed 3D chest computed tomography (CT) analyses revealed emphysema (low-attenuation area shown in red in CT images) in patients II-1 and II-2 (Goddard scores of 6 and 8, respectively) (Figure 9A) and multiple structural abnormalities in the bronchial tubes of patient I-1 (Figure 9B and Supplemental Figure 11), including deformity, dilatation, and thickening (Figure 9, C and D). However, the lung of patient I-1 did not exhibit emphysema. Patients II-1 and II-2 did not exhibit structural abnormalities in bronchial tubes.

### WGS analyses of LAMA5 region in all patients.

The somewhat subtle kidney phenotypes in the heterozygous KI mice prompted us to conduct WGS in the patients to examine whether intronic sequence alteration in the *LAMA5* region might underlie the phenotypic difference between the patients and heterozygous KI mice (Supplemental Table 3). We identified 12 minor variants commonly observed in our 3 patients (Table 1). They included g.60884421C>T, which was identified as a possible causative gene variant for this disease by the whole-exome analyses. Among these 12 variants, 11 were located in intronic regions but not in the promoter region of *LAMA5*. Among all 12 variants, we omitted 10 intronic variants because they demonstrated sufficiently high minor allele frequency (MAF) values in 8.3KJPN, gnomAD, or GEM-J WGA. Next, we omitted the remaining potentially novel intronic variant, chr20, g.60884695_60884695insGGGGGGGTGGGAGGGGGTGGG, because we also identified the same variant in the long read sequence data of genomic PCR derived from 3 healthy control genomes. The only remaining variant was g.60884421C>T.

## Discussion

A scaffolding protein, laminin α5, functions in a laminin complex as a ligand for integrins from adjacent cells. A patient with a homozygous pathogenic variant in the polymerization domain of laminin α5 was recently reported to demonstrate a wide-ranging phenotype, including nephropathy (15). However, genetic kidney diseases caused by heterozygous *LAMA5* variants have been difficult to identify, despite the recognition of several *LAMA5* variants associated with renal disease (18, 19). Here, we identified what we believe to be a novel heterozygous *LAMA5* variant, V3687M, as the gene variant possibly responsible for a renal disease: slowly progressive FSGS. Notably, corresponding heterozygous and homozygous KI mice also showed FSGS-like pathology and slight proteinuria. Consistent with our in vitro findings, both types of KI mice and our patient demonstrated reduced laminin α5 protein in GBM and bronchial BM, which is distinct from the findings for the recently reported homozygous variant *LAMA5* (15). However, owing to the lack of data on the secretion of full-length variant laminin α5, we could not definitively conclude that the reduced expression of laminin α5 in the GBM of the patient and KI mice was caused by the reduced secretion of variant protein from the adjacent cells.

The reduced expression of a ligand for integrins, laminin α5, implied that cell adhesion to BM might be compromised. We found that a mechanosensory protein in cell-matrix adhesions, vinculin, was enhanced in many components in glomeruli and bronchial epithelia, suggesting that the expression of this variant laminin α5 protein affects the mechanosensing of many cell types. Indeed, endothelial cell detachment was observed in patient glomeruli. By using podocyte-specific vinculin-KO mice, Lausecker et al. showed that vinculin is necessary for glomerular barrier integrity (24). Notably, endothelial cell–specific *Lama5*-KO mice exhibited reduced vinculin expression in vascular BM (25). Although the mechanism of vinculin enhancement was unclear in our model animals, the expression of variant laminin α5 protein in this disease has effects on cell-matrix adhesions that differ from the effects in *Lama5*-KO mice. Recently, Falcone et al. reported new NS model mice with the homozygous *Lama5* variant (11). Interestingly, their mice showed no obvious extrarenal phenotypes, although they also demonstrated decreased secretion of laminin α5 in GBM as in our KI mice. We proposed 2 possible explanatory mechanisms for these phenotypic discrepancies: 1) a difference in the ways in which laminin α5 protein is decreased between Lama5-KO mice and our disease model and 2) the toxic properties of secreted variant laminin α5 protein itself. To identify this mechanism, further investigation is needed. Furthermore, vinculin protein expression was reportedly decreased in glomeruli in patients with idiopathic FSGS or membranous nephropathy (24), suggesting that enhanced vinculin expression in glomeruli can differentiate this disease from other glomerulopathies before gene-specific analyses. In addition to vinculin enhancement in KI mice, we also identified the increased expression of ECM proteins (collagen I and fibulin 2) and the reduced expression of integrin β1, which were reported to be involved in the process of glomerulosclerosis (26) and primary FSGS (27), respectively. These findings suggest that the expression of variant laminin α5 in GBM causes a distinct glomerular injury in this disease.

In this study, we focused on compromised cell-matrix adhesion as a possible disease mechanism. However, the reduced secretion of variant laminin α5 may induce ER stress in cells. In vitro and in vivo analyses showed that the expression of an ER stress marker, GRP78, did not change with variant protein expression (Supplemental Figures 12 and 13). Thus, ER stress may not induce this disease, in contrast to the disease mechanisms in Pierson syndrome caused by a missense variant in the laminin β2 gene (28).

### Conclusion.

We discovered a possibly novel heterozygous variant in the *LAMA5* gene, which might lead to slowly progressive nephropathy with FSGS and pulmonary structural deformity, possibly through compromised cell adhesion. Enhanced expression of vinculin in affected tissues might be a useful disease marker.

### Limitations.

Regarding the limitations of this study, this variant, *LAMA5* V3687M, was found in only 1 pedigree, although we confirmed its responsibility for nephropathy with FSGS and lung deformity in heterozygous and homozygous KI mice. The compatible heterozygous KI mice also demonstrated milder renal phenotypes than our patients. Moreover, as another limitation, we analyzed the WGS only around the LAMA5 gene in the 3 patients, but we did not investigate other genome regions containing other genes causative of FSGS.

## Methods

### Whole-exome capture and sequencing.

For the 4 family members, the sequence libraries were prepared with SureSelect XT Human All Exon V6 (Agilent Technologies) and sequenced on a Hiseq 2500 (Illumina). After adapter trimming by cutadapt version 2.7, Burroughs Wheeler Alignment version 0.7.17 was used to align trimmed reads to the human reference sequence (GRCh37) and default parameters were used for fragment reads (29). Alignments were converted from SAM format to sorted and indexed BAM files by using SamTools (30) and Picard in GATK 4.1.2.0 (31). Picard was also used to remove invalid alignments and duplicate reads from BAM files. Base quality score recalibration was conducted using GATK BaseRecalibrator and ApplyBQSR. Genotypes were called using GATK HaplotypeCaller. Then, variants with variant quality score normalized by read depth less than 2.0, phred-scaled probability of strand bias greater than 60.0, root mean square of mapping quality of reads mapped to a nucleotide (MQ) less than 40.0, MQRankSum (normalized difference in mapping qualities between reference and alternative alleles) less than 12.5, ReadPosRankSum (normalized difference in position between reference and alternative alleles within the reads) less than 8.0, or strand bias calculated by symmetric OR test greater than 4.0 were filtered out by GATK VariantFiltration. Our microarray data were deposited in NCBI Gene Expression Omnibus database (accession number GSE215199).

Unions of single-nucleotide variant and indel variant calls from GATK were annotated with the ANNOVAR program to identify exonic or splicing variants, including their allele frequency and functional annotation (32). We analyzed the locations and genotypes of variants for each individual to locate subsets of variants on autosomal chromosomes. Output files from GATK were in VCF format. All variants were annotated using ANNOVAR2019Oct24 software with respect to their frequencies in the 1000 Genomes Project; National Heart, Lung, and Blood Institute Exome Sequencing Project (ESP); gnomAD; and 2 Japanese databases: 4.7KJPN in jMorp by Tohoku Medical Megabank Organization (ToMMo) and HGVD. Impacts on coding features were defined according to the following 3 databases: refGene tracks from the National Center for Biotechnology Information Reference Sequence Database, knownGene tracks from the University of California Santa Cruz Known Genes, and ensGene tracks from Ensemble Genes (32, 33). We selected candidate variants with the following criteria: 1) either an exonic or a splice site in at least 1 database; 2) nonsynonymous, splice site, insertion/deletion, stop gain/loss, or unknown variants in at least 1 database; 3) PASS in GATK annotation; and 4) an MAF of less than 0.1% in the total population in 1000 Genomes Project, ESP6500, gnomAD, 4.7KJPN, and HGVD.

### WGS on LAMA5 gene.

To determine the variants in the *LAMA5* gene in common among the 3 patients, WGS was performed on blood samples using the Illumina NovaSeq 6000. Adapter trimming, mapping, and variant calling were analyzed using the same method as for whole-exome sequencing. We selected candidate variants with the following criteria: 1) exonic and intronic regions on *LAMA5*; 2) PASS in GATK annotation; 3) an MAF of less than 0.5% in the total population in 1000 Genomes Project, ESP, gnomAD, 4.7KJPN (ToMMo), and HGVD. A variant not reported previously was confirmed by amplicon sequencing. PCR products were amplified with a primer set (forward: 5′ CTGGACAGGTGCTAGCGTGG 3′; and reverse: 5′ CCCTACCCTGACCCATCTCCT 3′) in 3 healthy individuals and sequenced by Illumina MiSeq. The data from WES and WGS of the patient family members are available in NBDC Human Database (study number JGAS000577).

### Development of V3687M-compatible KI mice.

C57BL/6JJcl mice were obtained from CLEA Japan. The animals were kept under conditions of 50% humidity and a 12-hour light/12-hour dark cycle. They were fed a standard pellet diet (MF; Oriental Yeast) and tap water ad libitum. We used the Alt-R CRISPR-Cas9 System for mouse genome editing (Integrated DNA Technologies). To design guide RNAs (gRNAs), software tools (http://crispor.tefor.net/ and https://crispr.dbcls.jp/) that predict unique target sites throughout the mouse genome were used. Single-stranded donor oligonucleotides (ssODN; 5′ CCCTGTCAACGTGACTGCTTCTGTACAAATCCAGGGGGCCATGGGGATGCGCGGATGCCCCTCAGGAACCCTAGCACTTTC 3′) were purchased from Integrated DNA Technologies. Pronuclear-stage mouse embryos were prepared by thawing frozen embryos (CLEA Japan) and cultured in KSOM medium (ARK Resource). For electroporation, 50–100 embryos at 1 hour after thawing were placed into a chamber with 40 μL of serum-free medium (Opti-MEM; Thermo Fisher Scientific) containing 100 ng/μL Cas9 protein, 200 ng/μL gRNA, or 300 ng/μL ssODN. They were electroporated with a 5 mm gap electrode (CUY505P5; Nepa Gene) in a NEPA21 Super Electroporator (Nepa Gene). Poring pulses for electroporation had the following parameters: voltage = 225 V, pulse width = 1 ms for mouse embryos, pulse interval = 50 ms, and number of pulses = 4. The first and second transfer pulses had the following characteristics: voltage = 20 V, pulse width = 50 ms, pulse interval = 50 ms, and number of pulses = 5. Mouse embryos that developed to the 2-cell stage after the introduction of gRNA and ssODN were transferred into the oviducts of female surrogates anesthetized with sevoflurane (Mylan N.V.). The resulting KI mice were genotyped by Sanger sequencing, which could differentiate heterozygous mice from homozygous or WT mice. The PCR primers for genotyping were as follows: Laminin α5 forward: 5′ GAGCCTGCCCAGTAAGTACG 3′; and Laminin α5 reverse: 5′ AAGGTCTGGGAACCCTCAAT 3′.

### Confirmation of the patient variant by Sanger sequencing.

Confirmation of the patient variant (chr20:60884421C>T) was performed by genomic PCR and Sanger sequencing. The PCR was performed using Tks Gflex DNA polymerase (Takara), in accordance with the manufacturer’s instructions. The primers for genomic PCR were as follows: forward: 5′ tcacaattaaaaatgggtggaagg 3′; and reverse: 5′ caccctcctatgatgtggaatgag 3′. For Sanger sequencing, BigDye Terminator Ready Reaction Mix (v3.1) (Thermo Fisher Scientific) was used. Then, the samples were purified using a BigDye XTerminator purification kit (Thermo Fisher Scientific).

### Prediction of variant pathogenicity.

The pathogenicity of the variant V3687M of *LAMA5* was evaluated by multiple functional prediction tools including SIFT (34), Polyphen2 (35), CADD (36), and PROVEAN (37).

### 3D structure prediction of control and mutant laminin α5 proteins.

3D structure prediction of control and mutant protein LG5 modules was conducted using the I-TASSER server (http://zhanglab.ccmb.med.umich.edu/I-TASSER/). The predicted 3D structure model with the highest reliability (C score: –0.19 [WT], –0.28 [V3687M]) was proposed.

### Staining of renal biopsy specimens.

Renal biopsy specimens were fixed in 10% neutral-buffered formalin and embedded in paraffin. Tissues were sectioned at 5 μm and stained using H&E, periodic acid–Schiff, and periodic acid methenamine silver protocols.

### Electron microscopy.

Tissues were fixed with 2% glutaraldehyde for 3 hours. After they had been transferred into 0.1 M phosphate buffer with 0.14 M sucrose (pH 7.4), tissues were embedded in resin. Transmission electron microscopy analyses were performed with an H7650 microscope (Hitachi).

### GBM thickness measurement.

GBM thicknesses in the electron microscopy images from control, patient II-2, WT mice, and both types of KI mice were measured using ImageJ software version 1.53m (NIH) (38). Sixty measurement points were randomly selected in each subject (39).

### Counting number of foot processes of podocyte per GBM length.

The number of foot processes per micrometer of GBM in the WT mice and both types of KI mice were quantified (24) using 10 mice at each time point. The length of GBM was measured using ImageJ software.

### IHC.

The paraffin tissue sections were clarified and incubated with Target Retrieval Solution (Dako) at 120°C for 15 minutes for antigen retrieval. After they had been washed 3 times with PBS, sections were blocked in Blocking One (Nacalai Tesque) for 30 minutes. The sections were then washed 3 times with PBS and incubated with primary Abs (Supplemental Table 4) for 1 hour at room temperature. After they had been washed 3 times with PBS, sections were incubated with the following secondary Abs at room temperature for 1 hour: donkey anti-rabbit IgG (H+L) Alexa Fluor 488, donkey anti-mouse IgG (H+L) Alexa Fluor 555, or donkey anti-goat IgG (H+L) Alexa Fluor 647 (Thermo Fisher Scientific). Both primary and secondary Abs were diluted with Can Get Signal Solution (Toyobo). The signals of Alexa Fluor 645 were converted to red in the figure. Nuclei were counterstained with DAPI (Wako Pure Chemical Industries). Sections were washed twice with PBS and then mounted with PermaFluor mounting medium (Thermo Fisher Scientific). Mouse tissues were frozen in Tissue-Tek optimum cutting temperature compound (Sakura Finetek). Sections were cut at 7 μm in a cryostat and air-dried. After blocking with 10% normal goat serum, the sections were incubated with primary Abs (Supplemental Table 4). Rabbit IgG was detected with secondary Ab conjugated with Alexa Fluor 594 (Thermo Fisher Scientific). Rat IgG and mouse IgG_1_ were detected with secondary Ab conjugated with Alexa Fluor 647 (Thermo Fisher Scientific). The signals of Alexa Fluor 647 were converted to green in the figure. After washing with PBS(–), sections were mounted in 90% glycerol containing 0.1 mL PBS and 1 mg/mL *p*-phenylenediamine. Sections were visualized under an LSM880 confocal microscope (Zeiss) and BZ-X800 (Keyence). Fluorescence intensity in the tissue was analyzed using Zen software (Zeiss).

### Measurement of fluorescence intensity of immunostaining of molecules in the glomeruli.

We randomly selected 7 different glomeruli from kidney tissue sections of WT and heterozygous KI mice and acquired images by confocal microscopy. In the analyses, we used the images from almost the same depths from the bottoms of sections. The mean fluorescence intensity was measured using Zen software. We repeated the same analyses using different sets of glomeruli in the tissue 3 times, obtaining almost the same statistical results.

### Abs and reagents.

All primary Abs used in this study are described in Supplemental Table 4. Human recombinant laminin-511 (LM511) and iMatrix-511 (LM511-E8) were purchased from BioLamina and Nippi, respectively.

### Construction of expression vectors.

DNA fragments encoding full-length rat laminin β2 were subcloned into the pIRESpuro3 Vector (Takara Bio). The expression vectors of mouse laminin α5 and γ1 used here were prepared in a previous study (40). The V3687M human laminin α5 mutation was engineered into analogous sites of the mouse cDNA by site-directed mutagenesis (QuikChange Site-Directed Mutagenesis Kit; Agilent Technologies).

The expression vector of Fc-SNAP-Tag (Fc-SNAP/pcDNAzeo) was prepared as follows. A DNA fragment encoding human IgG_1_ Fc and SNAP were amplified using primer sets described in Supplemental Table 5. CD4-Ig vector (41) and pSNAPf vector (New England Biolabs [NEB]) were used as templates for PCR. The PCR product was digested with XbaI and AvrII restriction enzymes (NEB), and DNA fragments encoding SNAP and Fc were sequentially inserted into the XbaI site of pcDNA3.1Zeo (+) (Thermo Fisher Scientific). The DNA fragments encoding human laminin γ2 signal peptide and LG4–5 modules of laminin α5 were also amplified using primer sets described in Supplemental Table 5. Full-length cDNA for WT and mutated laminin α5 was used as templates for PCR. The PCR products for human laminin γ2 signal peptide and laminin α5 LG4–5 modules were digested with KpnI/BamHI and BamHI/XbaI restriction enzymes (NEB), respectively. The DNA fragments were sequentially inserted into the corresponding restriction sites of Fc-SNAP/pcDNAzeo. The primer sequences used for making these constructs are shown in Supplemental Table 5.

### Preparation of serum-free conditioned media and cell lysates.

CHO-K1 cells were obtained from American Type Culture Collection. The cell line was maintained in DMEM (Merck) containing 10% fetal calf serum (Merck) and 70 μg/mL L-proline (Merck). To express WT and mutated α5LG4–5, the expression vectors were transiently transfected into the cells in 24-well plates using Lipofectamine 2000 (Thermo Fisher Scientific). The following day, transfected cells were labeled with SNAP-biotin (NEB) in accordance with the manufacturer’s protocol; the growth media were then replaced with 500 μL of serum-free medium. After 1 day of culture, the conditioned media were harvested and clarified by sequential centrifugation at 100*g* for 5 minutes and 9,100*g* for 10 minutes. Twenty microliters of conditioned medium were used for IB (described in the next section). Transfected cells were lysed with 500 μL of 10 mM Tris-HCl (pH 7.5), 150 mM NaCl, 10 mM EDTA, 1% NP-40, and protease inhibitor cocktail (Merck). Cell lysates were clarified by centrifugation at 9,100*g* for 10 minutes. Twenty microliters of cell lysate were used for IB (described in the next section).

### IB and quantitative analysis.

Cell lysates and conditioned media were separated on a 5%–20% gradient gel under reducing conditions and transferred to a PVDF membrane. Proteins on the membrane were detected using anti-human IgG Fc Ab (Jackson ImmunoResearch). Biotinylated SNAP-tagged proteins were probed with streptavidin HRP. Bound Abs and SNAP-tagged proteins were visualized with ECL Western Blotting Detection reagents (GE Healthcare). Images of bands were captured with WSE-6100 Lumino Graph I (ATTO) and quantified using ImageJ software. Total pixel density within a fixed square was measured on raw images, and mean band intensities were compared using Student’s *t* test.

### ELISA.

ELISA plates (96 well, Thermo Fisher Scientific) were coated with recombinant human laminin-511 and its E8 fragment and blocked with BSA in PBS(–). Diluted Abs were added and incubated at room temperature for 1 hour. After the plates had been washed with Tris-buffered saline with Tween 20, the bound Abs were detected using a HRP-conjugated anti-mouse IgG Ab followed by an addition of 0.4 mg/mL *o*-phenylenediamine and 0.01% H_2_O_2_. Absorbance was measured at 450 nm using a Multiskan GO Microplate Spectrophotometer (Thermo Fisher Scientific).

### Detailed 3D analyses of bronchial tubes using chest CT.

Chest CT examinations of patient 1 and an age- and sex-matched healthy control were conducted using a multidetector CT (Philips) under exactly the same conditions with informed consent. A total of 1,024 images per person, with a slice thickness of 0.7 mm on chest CT, were analyzed by SYNAPSE VINCENT software (Fujifilm). Seven segments of airways were measured at the position immediately prior to branching in terms of the difference between maximum and minimum inner diameter (maxDin-minDin, a marker of structural deformity), inner area, and mean thickness. We performed 5 measurements and statistically analyzed the data.

### Mouse urine albumin and urine creatinine quantification.

Albumin quantification in mouse urine was performed using an ELISA kit (AUTO WACO Micro Albumin; WAKO-Fujifilm), in accordance with the manufacturer’s instructions. Creatinine quantification in mouse urine was conducted using a measurement kit based on enzyme methods (L-type WACO CRE·M; WAKO-Fujifilm).

### Statistics.

The data were analyzed using the Mann-Whitney *U* test or 2-tailed Student’s *t* test in GraphPad Prism software (version 8; GraphPad). A *P* value less than 0.05 was considered significant. The error bars in the graphs represent SD.

### Study approval.

The human genome analysis research protocol was approved by the ethics committee of Osaka University (protocol approval number 618). Renal biopsy tissue from a healthy control volunteer was obtained from a 0-hour protocol biopsy of renal transplantation renal graft, after the provision of informed consent by that patient (Ethics Committee of Osaka University protocol approval number 08221). All animal experimental procedures were carried out in accordance with the guidelines for animal research approved by the animal research committee of Osaka University (protocol approval DOI00272).

## Author contributions

MN and YI conceived the study. JYK recruited patients and performed patient interviews. JYK and Y Kikkawa drafted the manuscript and performed the experiments. KH, YA, SK, YD, TO, YS, and KS generated the reagents or obtained tissue specimens from patients. TN-H performed pathology analyses. A Tanigawa, Y Kotani, YU, KY, and TM developed knockin mice. JYK, DM, A Takuwa, AO, KK, and AN performed data analyses.

## Supplementary Material

Supplemental data

## Figures and Tables

**Table 1:**
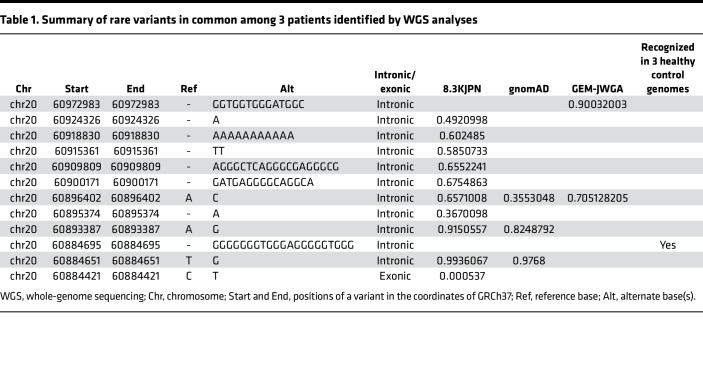
Summary of rare variants in common among 3 patients identified by WGS analyses

## Version 1 09/29/2022

In-Press Preview

## Version 2 12/08/2022

Electronic publication
