# Supplementary material for: A heterozygous LAMA5 variant may contribute to slowly progressive, vinculin-enhanced familial FSGS and pulmonary defects
Source: JCI Insight. 2022 Dec 8;7(23):e158378. doi: 10.1172/jci.insight.158378 (PMC9746903; doi:10.1172/jci.insight.158378)
Supplement: Supplemental data [file jciinsight-7-158378-s210.pdf]

Supplementary Figures and Tables of

A heterozygous *LAMA5* variant may contribute to slowly progerssive, vinculin-enhanced familial FSGS and pulmonary defects.

Running title: A heterozygous laminin α5 variant causes FSGS

Jun-Ya Kaimori[1,2*], Yamato Kikkawa[3*], Daisuke Motooka[4,5], Tomoko Namba-Hamano[2], Ayako Takuwa[5], Atsuko Okazaki[6,7], Kaori Kobayashi[6,8], Arisa Tanigawa[9], Yuko Kotani[9], Yoshihiro Uno[9], Kazuto Yoshimi[10,11], Koki Hattori[2], Yuta Asahina[2], Sachio Kajimoto[2], Yohei Doi[2], Tatsufumi Oka[2], Yusuke Sakaguchi[1,2], Tomoji Mashimo[9,10,11,12], Kiyotoshi Sekiguchi[13], Akihiro Nakaya[6,14], Motoyoshi Nomizu[3], and Yoshitaka Isaka[2]

[1]Department of Inter-Organ Communication Research in Kidney Diseases, Osaka University Graduate School of Medicine

[2]Department of Nephrology, Osaka University Graduate School of Medicine

[3]Department of Clinical Biochemistry, Tokyo University of Pharmacy and Life Sciences

[4]Genome Information Research Center, Research Institute for Microbial Diseases

[5]Immunology Frontier Research Center, Osaka University

[6]Department of Genome Informatics, Osaka University Graduate School of Medicine

[7]Diagnostics and Therapeutics of Intractable Diseases, Intractable Disease Research Center, Graduate School of Medicine, Juntendo University

[8]Medical Solutions Division, NEC Corporation

[9]The Institute of Experimental Animal Sciences, Osaka University Graduate School of Medicine

[10]Genome Editing Research and Development (R&D) Center, Osaka University Graduate School of Medicine

[11]Division of Animal Genetics, Laboratory Animal Research Center, Institute of Medical Science

[12]Division of Genome Engineering, Center for Experimental Medicine and Systems Biology, Institute of Medical Science

[13]Division of Matrixome Research and Application, Institute for Protein Research

[14]Laboratory of Genome Data Science, Graduate School of Frontier Sciences, The University of Tokyo

*Corresponding authors

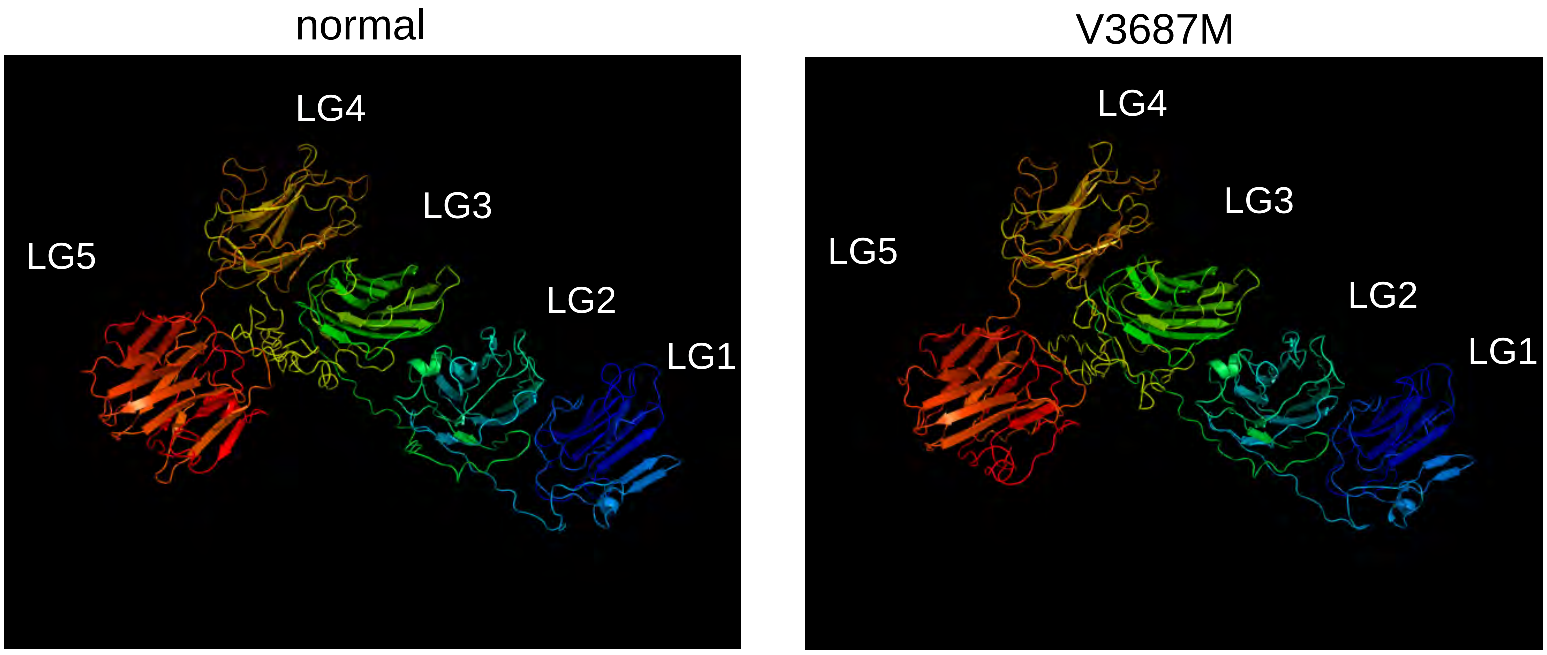

Supplementary Figure S1. Predicted 3D structures of normal, the variant V3687M laminin α5 LG1-5 module.

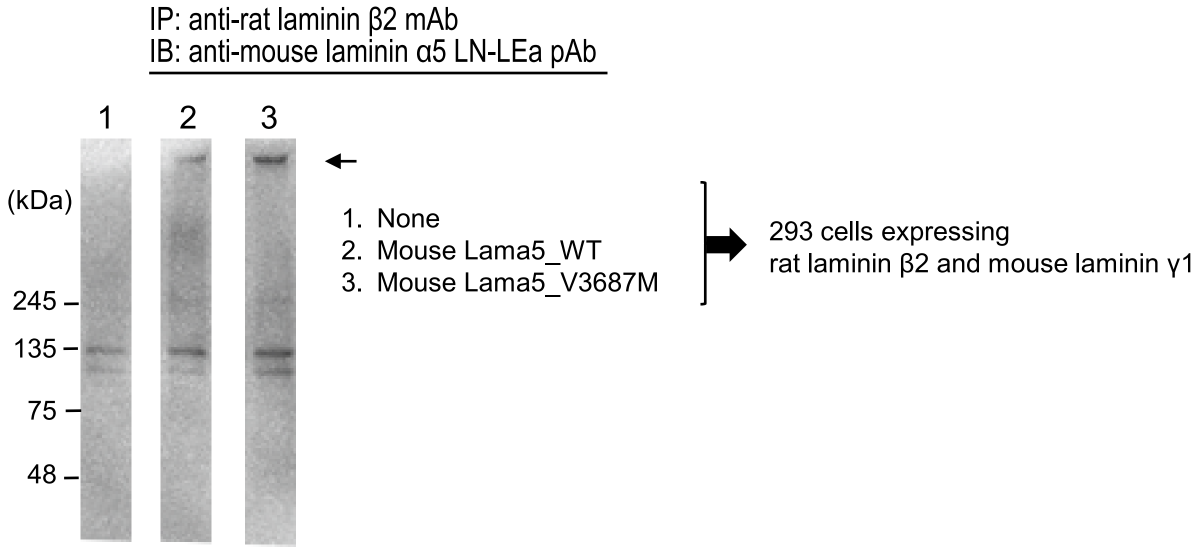

Supplemenary Figure S2. Assembly of mutant proteins into laminin heterotrimers.
Expression vectors of full-length wild-type (lane 2) and mutant laminin α5 V3687M (lane 3), G3685R were transiently transfected into the HEK293 cells expressing β2 and γ1 chains. Negative control (lane 1). The conditioned media from the cells were used for immunoprecipitation with anti-rat laminin β2 monoclonal antibody. The immunoprecipitated proteins were separated on 5-20% gel under non-reducing condition and transferred to PVDF membrane. The immune complexes were detected with anti-mouse laminin α5 domain LN/LEa polyclonal antibody, as described in following Experimental procedures. The mutant laminin α5 chain assembled with β2 and γ1 chains as well as wild-type.

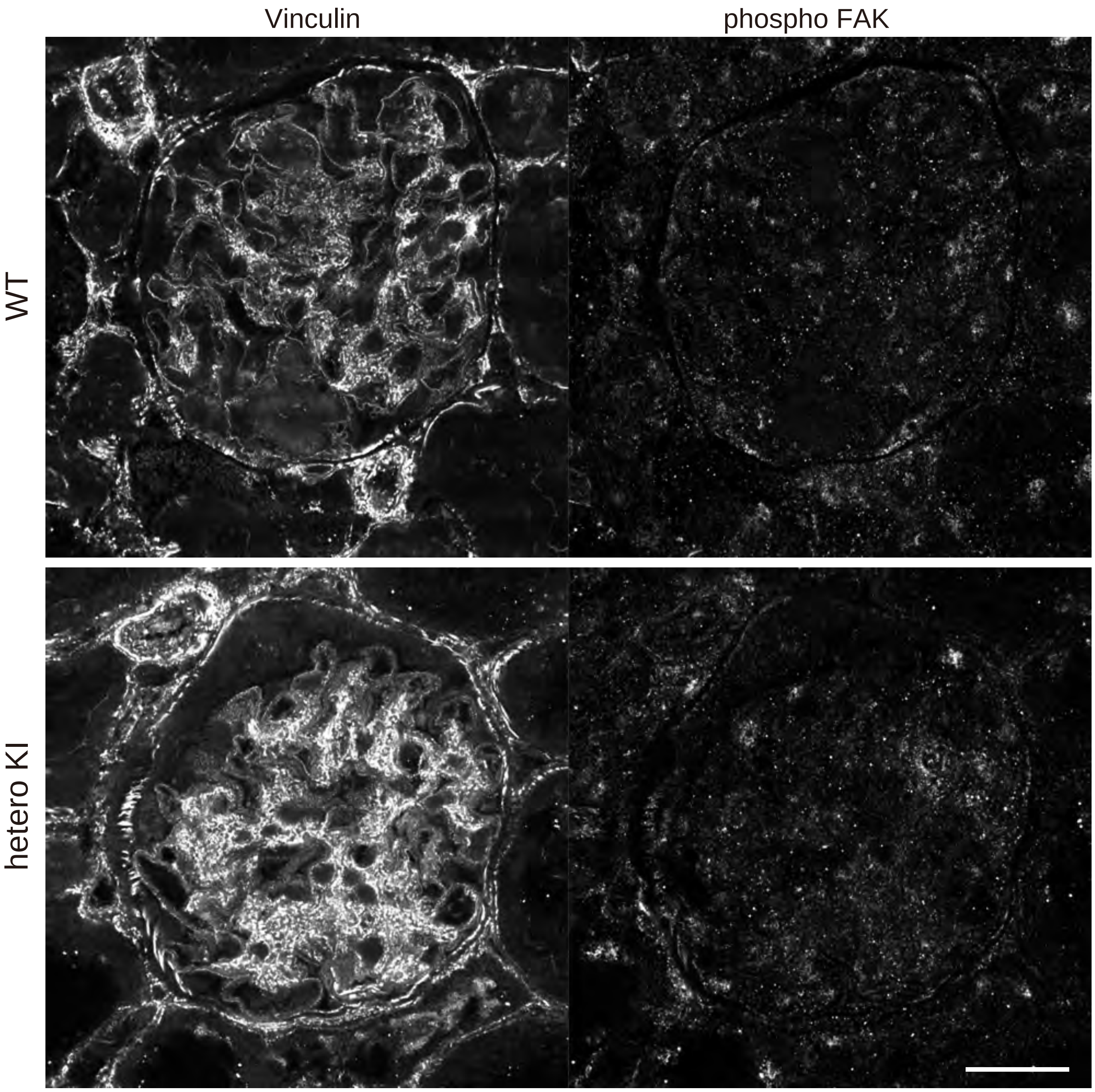

Supplemenatry Figure S3. Immunofluorescent staining of vinculin and phosphoFAK in kidney glomeruli from WT and heterozygous KI mice. Bar: 20 μm.

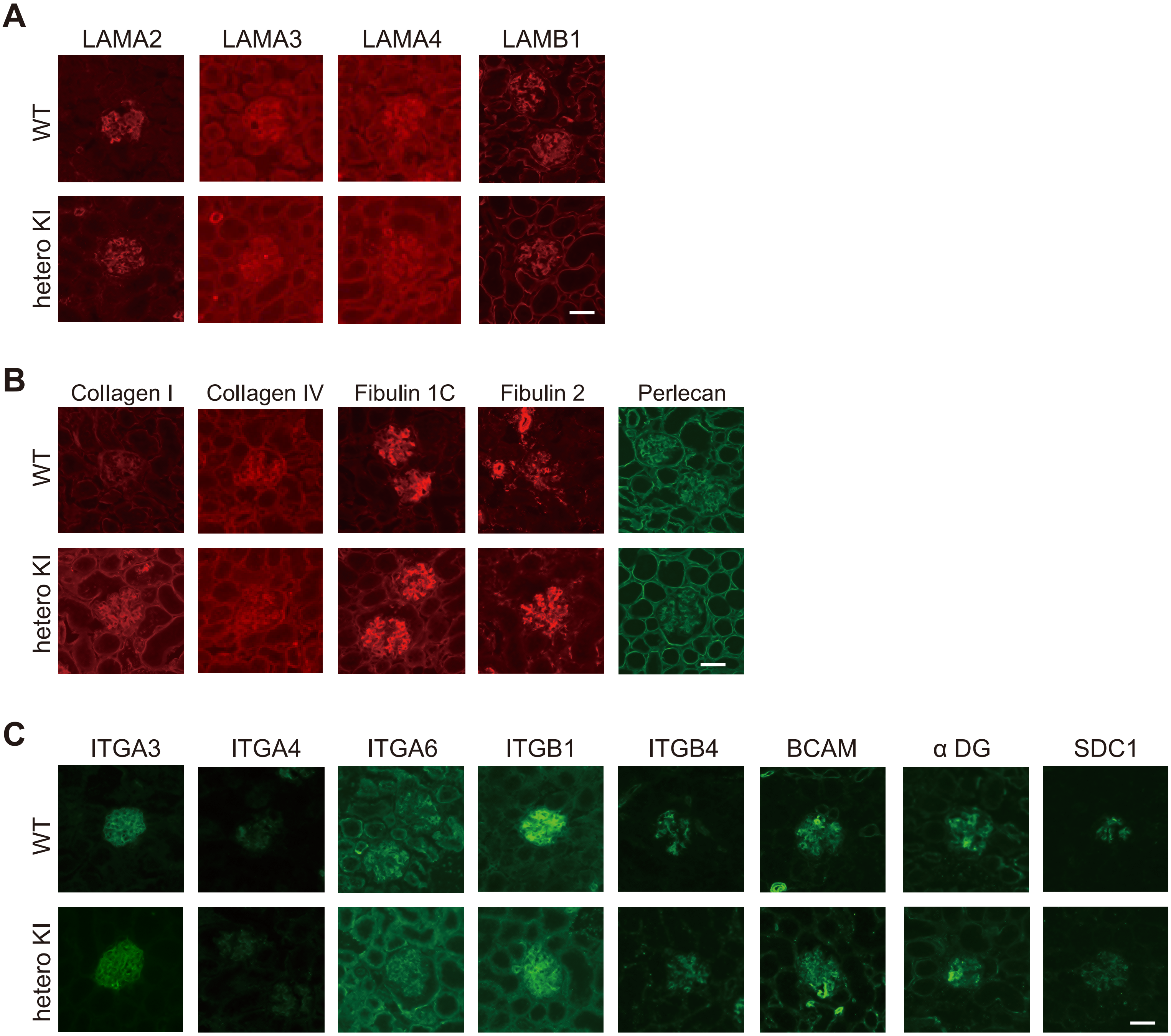

Supplementary Figure S4. Immunofluorescence staining images of other chains of laminin, other components of extra cellular matrix and receptors for laminin α5 in kidney. Immunofluorescence staining images of chanis of laminin **(A)**, components of ECM **(B)** and receptors for laminin α5 **(C)** in kidney glomeruli from WT and heterozygous KI mice. Bar: 20 µm.

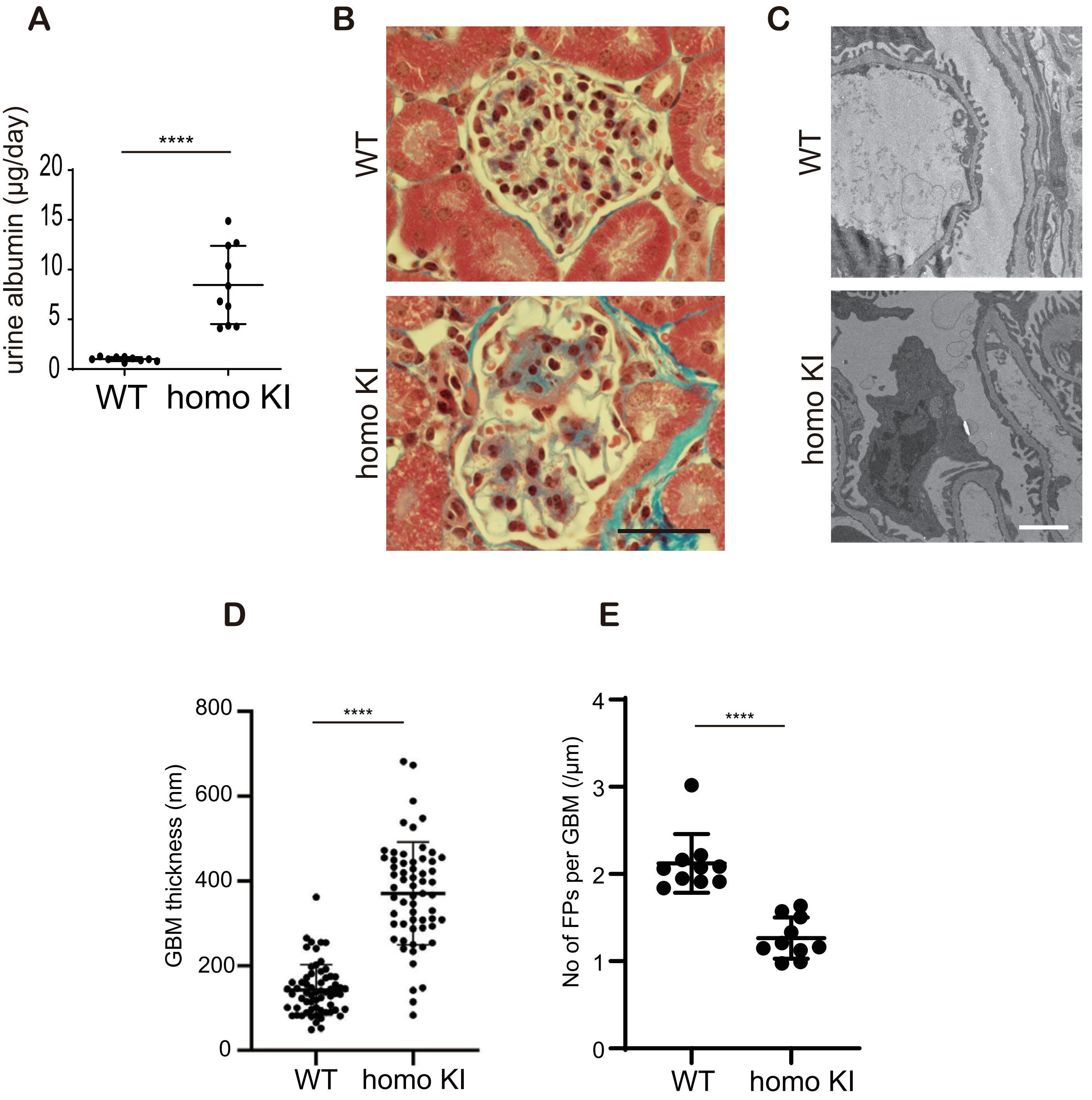

Supplementary Figure S5. Renal phenotypes of homozygous V3684M KI mice at 16 weeks of age. (A) Urine albumin excretion per day of WT and homozygous V3684M KI mice at 16 weeks of age. ****: $p<0.0001$; Mann–Whitney U test. **(B)** Masson’s trichrome staining images of kidney tissues from WT and V3684M KI mice. Bar: 20μm. **(C)** Electron micrographs of GBM from WT and heterozygous KI mice. Bar: 2 μm. **(D)** GBM thickness of WT and homozygous V3684M KI mice at 16 weeks of age. ****: $p<0.0001$; Mann–Whitney U test. **(E)** Number of foot processes per GBM length in WT and homozygous V3684M KI mice at 16 weeks of age.****: $p<0.0001$; Mann–Whitney U test.

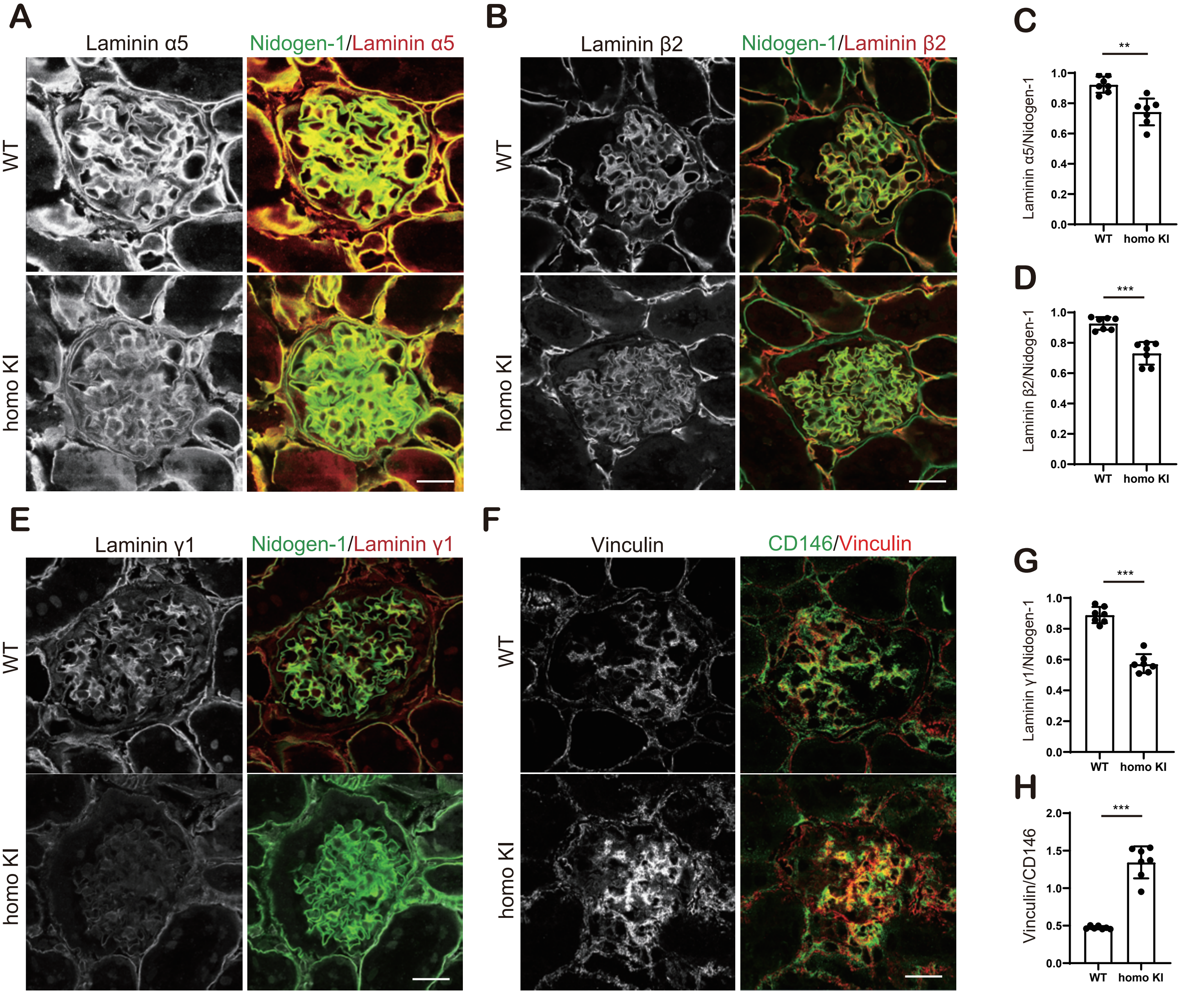

Supplementary Figure S6. Protein expression of laminin α5, β2, γ1 and vinculin in the glomeruli in homozygous V3684M KI mice at 16 weeks of age. Immunofluorescence staining images of laminin α5 **(A)**, β2 **(B)**, γ1 **(E)** and nidogen-1 in kidney glomeruli from WT and homozygous KI mice. Bar: 20 µm. Quantification of laminin α5 **(C)**, β2 **(D)**, γ1**(G)** relative to nidogen-1 intensity in the glomerular area. Mean fluorescence intensity of each stain was calculated by Zen software. **: p<0.01, ***: p<0.001; Mann–Whitney U test. **(F)** Immunofluorescence staining images of CD146 and vinculin in kidney glomeruli from WT and homozygous KI mice. Bar: 20 µm. **(H)** Quantification of vinculin relative to CD146 intensity in the glomerular area. ***: p<0.001; Mann–Whitney U test.

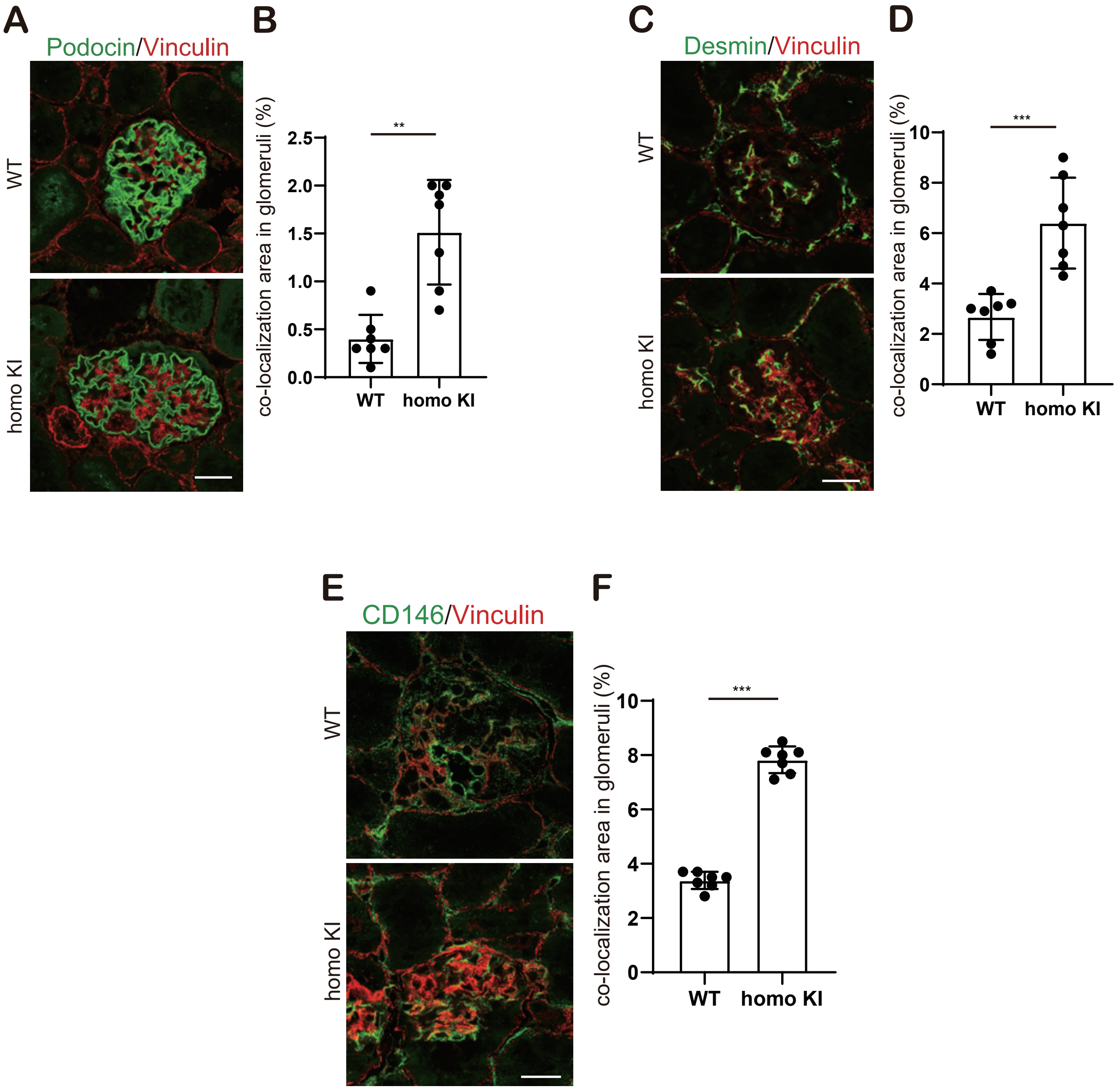

Supplementary Figure S7. Increased vinculin localization in various glomerular components of homozygous V3684M KI mice at 16 weeks of age. (A) Immunofluorescence staining images of podocin and vinculin in kidney glomeruli from WT and homozygous KI mice. Bar: 20 µm. **(B)** Quantification of vinculin and podocin co-localization area per glomerular area. **: $p<0.01$; Mann–Whitney U test. **(C)** Immunofluorescence staining images of desmin and vinculin in kidney glomeruli from WT and homozygous KI mice. Bar: 20 µm. **(D)** Quantification of vinculin and desmin co-localization area per glomerular area. ***: $p<0.001$; Mann–Whitney U test. **(E)** Immunofluorescence staining images of CD146 and vinculin in kidney glomeruli from WT and homozygous KI mice. Bar: 20 µm. **(F)** Quantification of CD146 and desmin co-localization area per glomerular area. ***: $p<0.001$; Mann–Whitney U test.

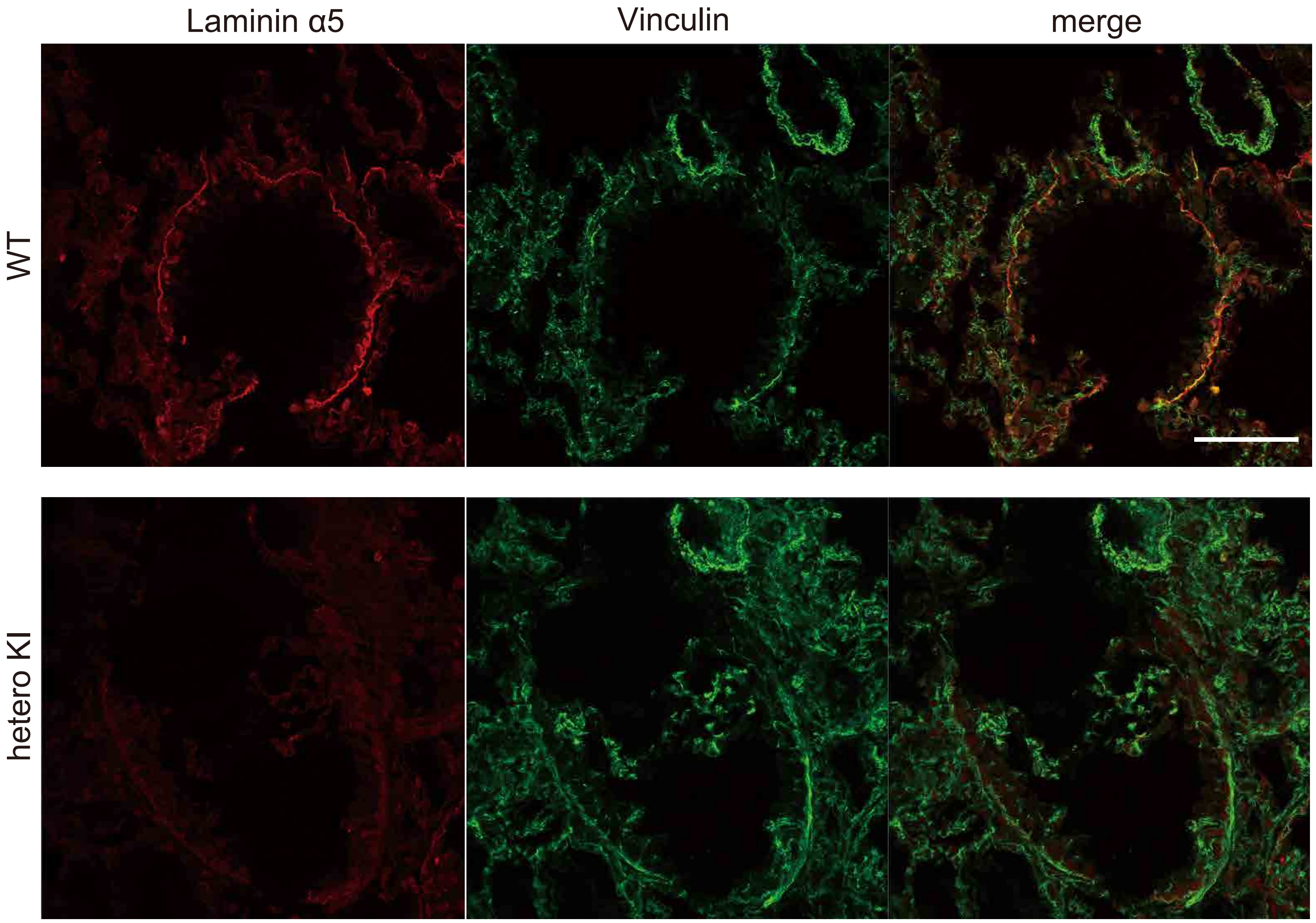

Supplementary Figure S8. Co-immunostaining of laminin α5 and vinculin in lung tissues from WT and heterozygous KI mice. The reduction of laminin α5 and the increase of vinculin in the lung tissue from a heterozygous mouse were observed in the merged images. Bar: 50 μm.

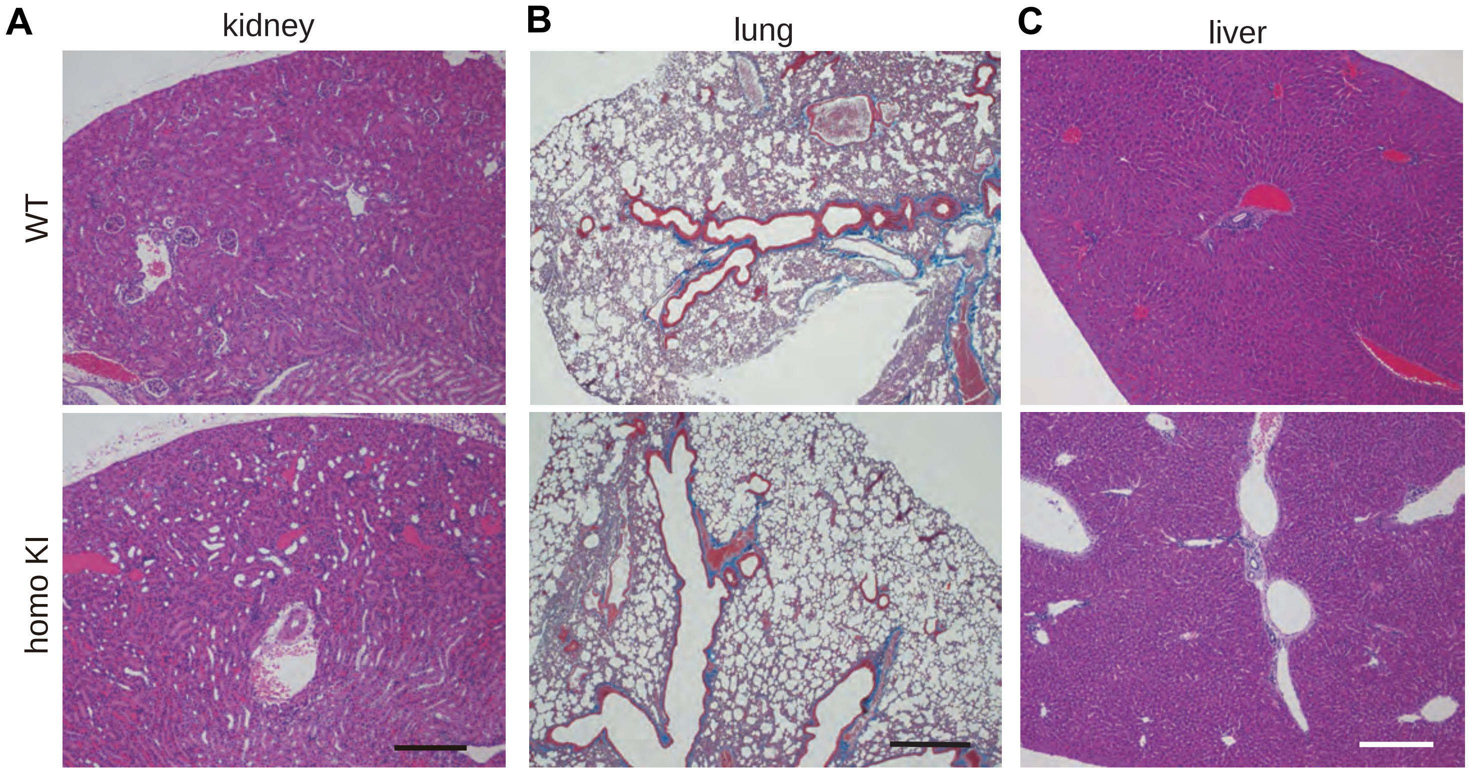

Supplementary Figure S9. Systemic phenotypes of homozygous V3684M KI mice at 16 weeks of age. (A) Lower magnified images of HE staining of kdiney tissues form WT and homozygous V3684M KI mice at 16 weeks of age. Delated tubules and veins were observed. Bar:1000 μm.**(B)** Lower magnified images of Masson's trichrome staining of lung tissues from WT and heterozygous V3684M KI mice. Delated bronchial tubes and enlarged alveolar area were observed in the lung tissue from homozygous KI mice at 16 weeks of age. Bar: 1000 μm.**(C)** Lower magnified images of HE staining of liver tissues form WT and homozygous V3684M KI mice at 16 weeks of age. Delated veins were observed. Bar:1000 μm.

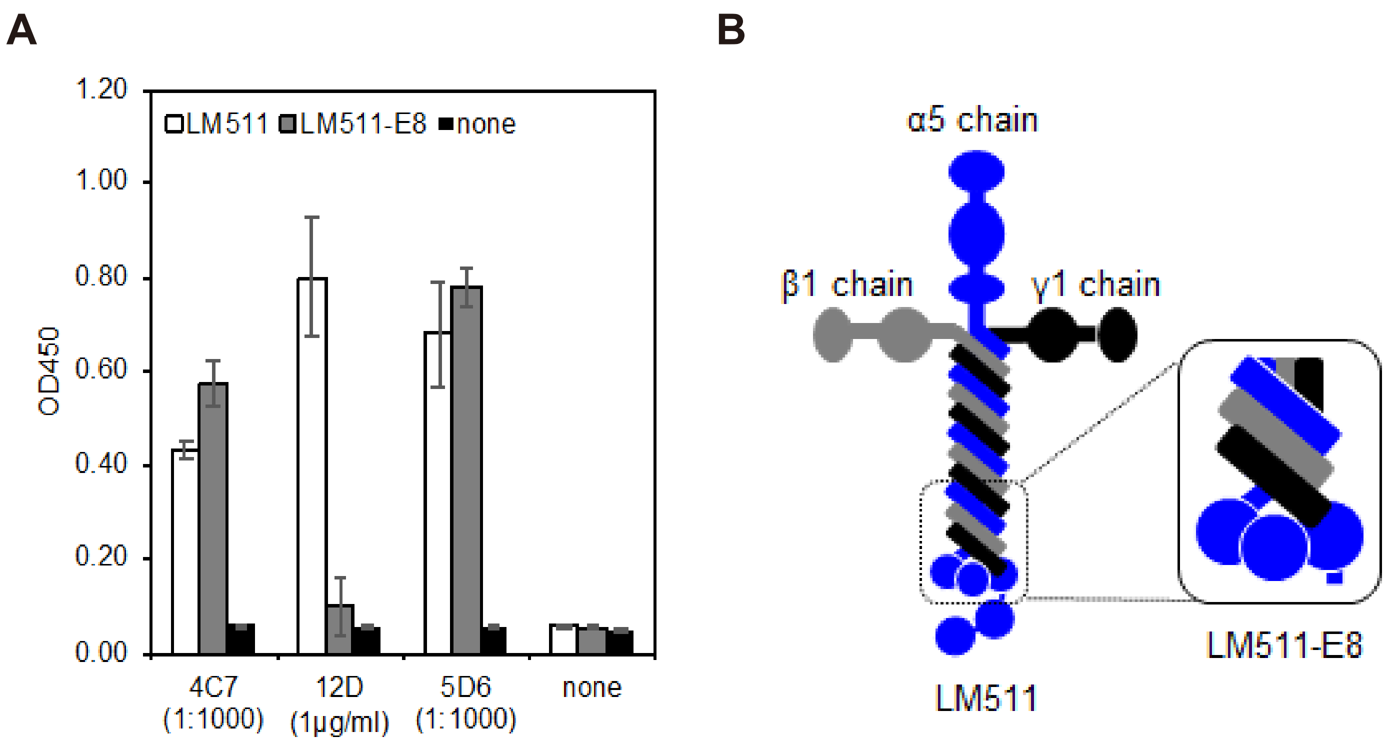

Supplemental Figure S10. Epitope mapping of monoclonal antibodies against human laminin α5 chain. (A) Ninety-six-well ELISA plates were coated with whole molecule and E8 fragment of laminin-511.ELISA demonstrates that the epitopes of 5D6 and 4C7 monoclonal antibodies were localized in the E8fragment of laminin-511. The 5D6 antibody is not recognized α5LG4-5 in which the mutation is located.**(B)** The schematic presentation of LM511 molecule.

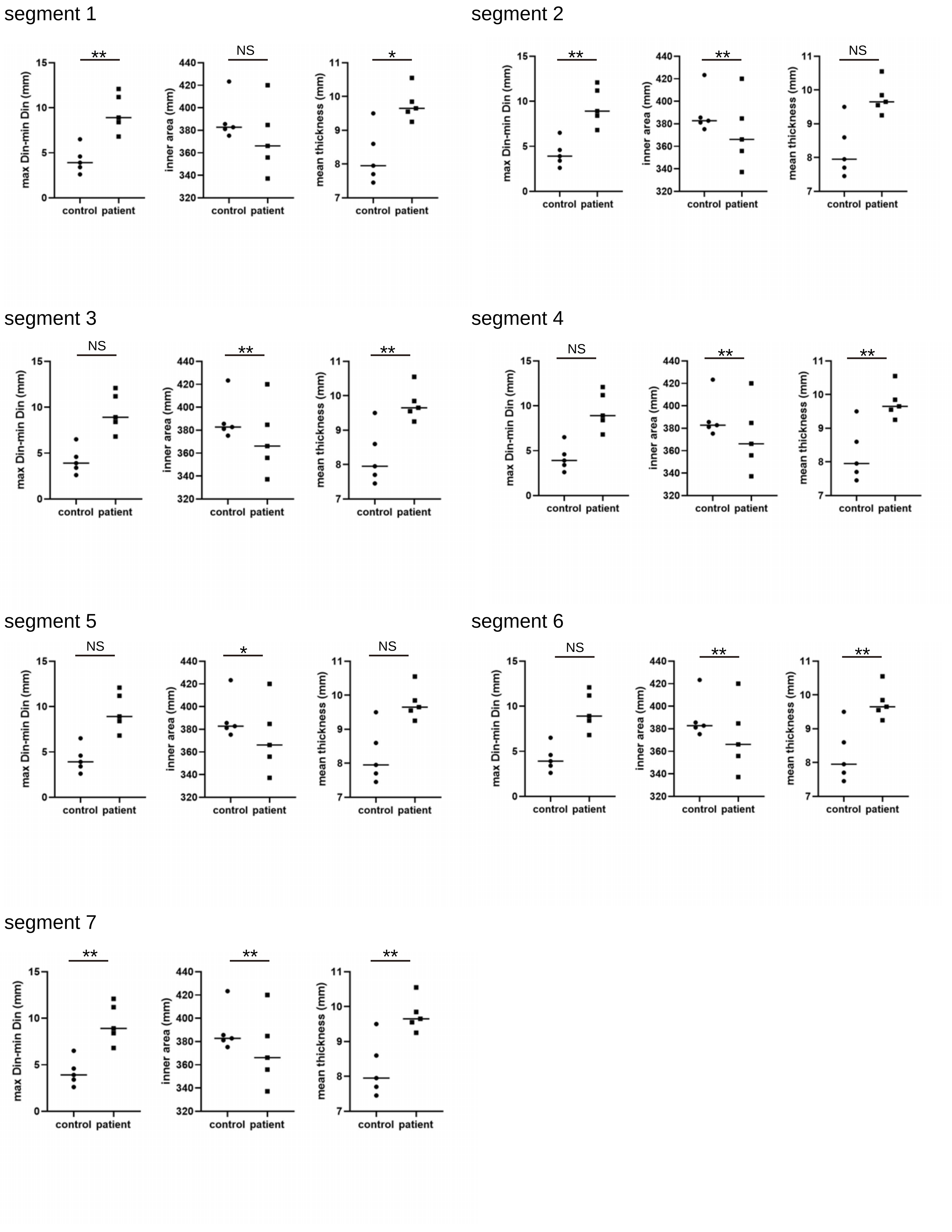

Supplemental Figure S11. Measured data and stastical analyses in detailed 3D analyses of bronchial tubes using patient chest CT. NS: not significant. *: $P<0.05$, **$P<0.01$, Mann-Whitney test.

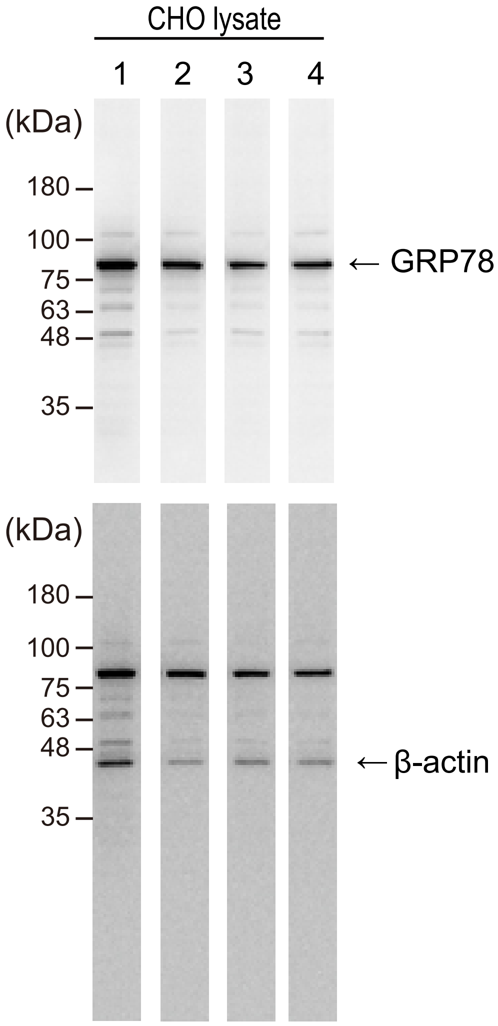

Supplementary Figure S12. Expression of GRP78 in CHO-K1 transfectants.

Upper panel:Cell lysates of transfectants expressing wild type (lane 3) and Mutant V3687M (lane 4) α5LG4-5/Fc-SNAP fusion proteins were applied to immunoblot analysis. Negative control and Fc-SNAP protein (lane 1 and 2). Lower panel:β-actin was used as internalcontrol.

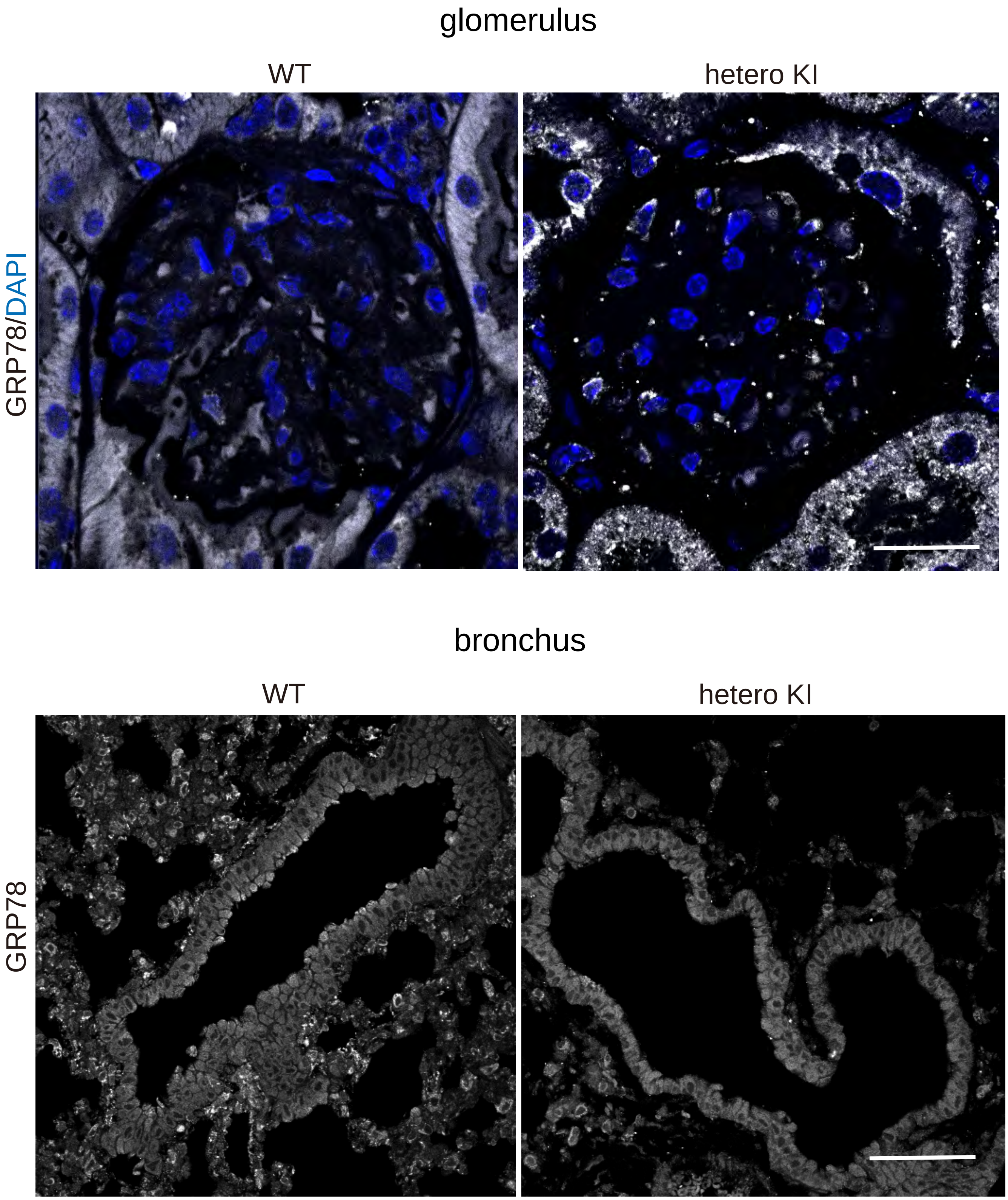

Supplementary Figure S13. ER stress marker, GRP78 staining in WT and heterozygous V3684M KI mice glomeruli and bronchial epitherium.
Bars: 20 µm.

Supplementary Table S1. Whole exome sequence data summary.

	I-1	I-2	II-1	II-2	all
average of DP in 54 variants	80.89	40.04	69.30	76.07	66.6
raw data(bp)	3,666,827,500	3,829,399,100	3,248,050,900	3,459,781,800	
trimmed data (bp)	3,663,285,817	3,825,621,819	3,244,881,459	3,456,358,232	
%mapped reads(paired)	99.93%	99.93%	99.94%	99.93%	
called variant number per sample	458373	468753	423643	444353	
rare variant number (MAF<0.1%) per sample	408	440	491	461	
patient unique rare variant number (MAF<0.1%)	54				

Supplementary Table S2. The 49 candidate genes with 54 rare variants (MAF<0.1%) unique to patients.

MAST2	HNRNPM
ABCA4	IQCN
S100A7A	DMKN
NES	HRC
EDEM3	MYH14
TPO	ZNF534
BABAM2	SLX4IP
ZNF806	NKX2-2
TTN	RIPOR3
GLS	LAMA5
COPS9	ASMTL
CROCC2	ARSD
PIGG	LITAF
MFSD10	PIEZO1
LINC01096	AMDHD2
ADGRV1	PTPRF
MZB1	SEMA6B
NT5C3A	
SEMA3C	
MTMR9	
ZC3H3	
MYORG	
LOC103908605	
RAD23B	
LAMC3	
RXRA	
RBM20	
PTPRE	
GUCY2EP	
DGKH	
SPTBN5	
APH1B	
AMDHD2	

Supplementary Table S3. Whole genome sequence data summary.

	I-2	II-1	II-2
raw data (bp)	110,618,535,986	110,807,157,636	112,581,321,566
trimmed data (bp)	109,623,934,816	109,702,226,167	111,321,817,446
%mapped reads (paired)	98.8%	98.9%	98.8%
called variant number per sample	5118967	5131260	5152632
called variant number per sample on *LAMA5*	234	216	225
rare variant number (MAF<0.5%) per sample on *LAMA5*	14	12	13
patient unique rare variant number (MAF<0.5%) on *LAMA5*	12		

Antibody to	Epitope	Clone/Lot	Host/Antigen Species	Ig class	Source/Reference	Application
Laminin α1	LN/LEa domain	1057+	Rabbit/Mouse	-	[1]	IHC
Laminin α2	LG domain	α2G	Rabbit/Human	-	[2]	IHC
Laminin α3	LEc domain	1110+	Rabbit/Mouse	-	[3]	IHC
Laminin α4	LEc domain	1129+	Rabbit/Mouse	-	[4]	IHC
Laminin α5	LEb/L4b	8948	Rabbit/Mouse	-	[5]	IHC
Laminin α5	E8 fragment	5D6	Mouse/human	-	[6]	IHC
Laminin β1	LF domain	1065+	Rabbit/Mouse	-	Sasaki T et al, Eur J Biochem, 2002	IHC
Laminin β1	unknown	2AB1-IA10	Rat/Mouse	purified IgG	ATCC, Manassas, VA	IHC
Laminin β2	LF domain	1117+	Rabbit/Mouse	-	[7]	IHC
Laminin γ1	LN/LEa domain	1083+	Rabbit/Mouse	-	Sasaki T, Oita University	IHC
Perlecan	unknown	A7L6	Rat/Mouse	IgG2a	abcam, Cambridge, UK	IHC
Nidogen-1	unknown	ELM1	Rat/Mouse	IgG2a	Merck, Kenilworth, NJ	IHC
Fibulin-1C	unknown	1034+	Rabbit/Mouse	-	[8]	IHC
Fibulin-2	unknown	1028+	Rabbit/Mouse	-	[8]	IHC
Collagne I	unknown	ab34710	Rabbit/Human and bovine	-	abcam, Cambridge, UK	IHC
Collagne IV	unknown	ab6586	Rabbit/Human and bovine	-	abcam, Cambridge, UK	IHC
Collagen IVα5	SKPQSETL	H52	Rat/Human	-	Chrondrex,Woodinville, WA	IHC
Integrin α3	Extracellular domain	2A10	Rat/Mouse	IgG2a	Mekada E, Osaka University	IHC
Integrin α4	Extracellular domain	R1-2	Rat/Mouse	IgG2b	BioLegend, San Diego, CA	IHC
Integrin α6	Extracellular domain	GoH3	Rat/Mouse	IgG2a	BD Bioscience, Franklin Lakes, NJ	IHC
Integrin β1	Extracellular domain	1D7	Rat/Mouse	IgG2a	Mekada E, Osaka University	IHC
Integrin β4	Extracellular domain	346-11A	Rat/Mouse	IgG2a	BD Bioscience, Franklin Lakes, NJ	IHC
Lutheran/BCAM	Extracellular domain	10-5	Rat/Mouse	IgG2a	[9]	IHC
α-dystroglycan	Extracellular domain	3D7	Rat/Mouse	IgG2a	Merck, Kenilworth, NJ	IHC
Syndecan-1	Extracellular domain	281-2	Rat/Mouse	IgG2a	BioLegend, San Diego, CA	IHC
Desmin	unknown	D33	Mouse/Human	IgG1	Santa Cruz, Dallas, TX	IHC
Desmin	C-terminus	D93F5	Rabbit/Human	purified IgG	CST, Danvers, MA	IHC

Vinculin	unknown	hVIN-1	Mouse/Human	IgG1	Merck, Kenilworth, NJ	IHC
Podocin	C-terminus	ab50339	Rabbit/Mouse	-	abcam, Cambridge, UK	IHC
Podocin	C-terminus	HPA049486	Rabbit/Human	-	Merck, Kenilworth, NJ	IHC
CD146	Extracellular domain	m146	Rabbit/Mouse	-	[10]	IHC
Bip/GRP78	C-terminus	-	Rabbit/Mouse	-	abcam, Cambridge, UK	IB
Bip/GRP78	N-terminus	N20	Goat/Human	-	Santa Cruz, Dallas, TX	IHC

Supplementary Table S4. Primary antibodies used in this research.

IHC: immunohistochemistry; IB: immunoblotting

References

1. Ettner N, Gohring W, Sasaki T, *et al.* The N-terminal globular domain of the laminin alpha1 chain binds to alpha1beta1 and alpha2beta1 integrins and to the heparan sulfate-containing domains of perlecan. *FEBS Lett* 1998; **430:** 217-221.

2. Cheng YS, Champliaud MF, Burgeson RE, *et al.* Self-assembly of laminin isoforms. *J Biol Chem* 1997; **272:** 31525-31532.

3. Tunggal L, Ravaux J, Pesch M, *et al.* Defective laminin 5 processing in cylindroma cells. *Am J Pathol* 2002; **160:** 459-468.

4. Sasaki T, Mann K, Timpl R. Modification of the laminin alpha 4 chain by chondroitin sulfate attachment to its N-terminal domain. *FEBS Lett* 2001; **505:** 173-178.

5. Miner JH, Patton BL, Lentz SI, *et al.* The laminin alpha chains: expression, developmental transitions, and chromosomal locations of alpha1-5, identification of heterotrimeric laminins 8-11, and cloning of a novel alpha3 isoform. *J Cell Biol* 1997; **137:** 685-701.

6. Fujiwara H, Kikkawa Y, Sanzen N, *et al.* Purification and characterization of human laminin-8. Laminin-8 stimulates cell adhesion and migration through alpha3beta1 and alpha6beta1 integrins. *J Biol Chem* 2001; **276:** 17550-17558.

7. Sasaki T, Mann K, Miner JH, *et al.* Domain IV of mouse laminin beta1 and beta2 chains. *Eur J Biochem* 2002; **269:** 431-442.

8. Pan TC, Sasaki T, Zhang RZ, *et al.* Structure and expression of fibulin-2, a novel extracellular matrix protein with multiple EGF-like repeats and consensus motifs for calcium binding. *J Cell Biol* 1993; **123:** 1269-1277.

9. Miura Y, Matsui S, Miyata N, *et al.* Differential expression of Lutheran/BCAM regulates biliary tissue remodeling in ductular reaction during liver regeneration. *Elife* 2018; **7**.

10. Hamano N, Kamoshida S, Kikkawa Y, *et al.* Development of Antibody-Modified Nanobubbles Using Fc-Region-Binding Polypeptides for Ultrasound Imaging. *Pharmaceutics* 2019; **11**.

Protein	Primer	Sequence (5'-3')
SNAP-Tag	SNAP01	GGAATTCGTCTAGACATGGACAAAGACTGCGAAATG
	SNAP02	CGCCTAGGCTAACCCAGCCCAGGCTTGCCCAG
Human IgG$_1$ Fc-Tag	Fc01'	GAGGATCCCGAGGGTGAGTACTAA
	Fc04	GACTCCTAGGCCTTTACCCGGAGACAGGGAGAGGCT
Signal sequence of human laminin γ2 chain	HG2SIG01	GGGGTACCGCCATGCCTGCGCTCTG
	HG2SIG02'	CGGGATCCGTCGACGCTAGCACTTCCCTCCTGGAGGTGGC
Mouse laminin α5LG4-5 modules	LA5G09	GCTCTAGAGCCCAGCCAGGACCTTGCCTGC
	LA5G18	GGGATCCGTTCCTGAGGGGCATCCGCGCAT

Supplementary Table S5. Primer sets.
